# Ultrastructure and functional morphology of the appendages in the reef-building sedentary polychaete *Sabellaria alveolata* (Annelida, Sedentaria, Sabellida)

**DOI:** 10.1186/s40850-021-00068-8

**Published:** 2021-03-09

**Authors:** Christian Meyer, Thomas André, Günter Purschke

**Affiliations:** Department of Biology and Chemistry, Zoology and Developmental Biology, University of Osnabrück, Barbarastr. 11, 49069 Osnabrück, Germany

**Keywords:** Tentacular filaments, Palps, Opercular papillae, Branchiae, Blood vessels, Coelom, Operculum, Receptor cells, Cartilage tissue, Ciliated epithelia

## Abstract

**Background:**

The sedentary polychaete *Sabellaria alveolata*, the sandcastle or honeycomb worm, possesses four different kinds of appendages besides the parapodia: opercular papillae, tentacular filaments, palps, and branchiae. It exhibits a highly specialized anterior end, the operculum, formed by the prostomium, peristomium, and two anterior segments. The operculum comprises opercular papillae, tentacular filaments, and palps. Paired branchiae are present from the second thoracic chaetiger onwards on the posteriorly following segments except for the last ones. Ultrastructural data on these appendages are either scanty, incomplete, or even lacking in Sabellariidae. In order to analyze their functional morphology, to bridge the data gap, and providing data for future phylogenetic and evolutionary analyses, we investigated the appendages of *S. alveolata* by applying light microscopy, confocal laser scanning microscopy, scanning, and transmission electron microscopy.

**Results:**

In *S. alveolata* the entire body is covered by a thin cuticle characterized by the absence of layers of parallel collagen fibers with no differentiation between the various body regions including the branchiae. The opercular papillae bear numerous tufts of receptor cells and lack motile cilia. The tentacular filaments show a distinctive pattern of motile cilia. Their most conspicuous morphological feature is a cell-free cartilaginous endoskeletal structure enclosed by ECM. Besides musculature the filaments include a single coelomic cavity but blood vessels are absent. The palps are ciliated and possess two coelomic cavities and a single blind-ending internal blood vessel. Besides external ciliation and receptor cells, the coelomate branchiae are highly vascularized and equipped with numerous blood spaces extending deep between the epidermal cells resulting in low diffusion distances.

**Conclusions:**

All appendages, including the branchiae, bear receptor cells and, as such, are sensory. The opercular papillae resemble typical parapodial cirri. In contrast, the tentacular filaments have a triple function: sensing, collecting and transporting particles. A similarity to branchiae can be excluded. The palps are typical grooved palps. A revised classification of polychaete branchiae is suggested; thereby, the branchiae of *S. alveolata* belong to the most common type comprising coelom, musculature, and blood vessels. The results indicate that diffusion distances between blood and environment have been underestimated in many cases.

**Supplementary Information:**

The online version contains supplementary material available at 10.1186/s40850-021-00068-8.

## Background

Sabellariidae Johnston, 1865 is a highly specialized group of sedentary annelids living in tubes built of cemented sand grains [1–3]. Although the group only comprises approximately 130 species, they are well known because of the gregarious behavior of certain species that may form large reefs. Due to the characteristic shape of their tube openings, they are also known as sandcastle or honeycomb worms. The animals exhibit heteronomous segmentation, and their highly modified anterior end forms the operculum representing a multifunctional structure [3–5]. Their body may be subdivided into operculum, thorax, parathorax, abdomen and caudal region [2–4, 6]. Sometimes the thorax is included in the operculum proper. Besides the parapodia, the animals bear four different kinds of appendages, opercular papillae, tentacular filaments, palps, and branchiae. Whereas the former three are related to the operculum, the branchiae are present for multiple segments beginning with the second thoracic segment [3].

The operculum is the most characteristic feature of sabellariids and represents the head region, which seals the tube when the animals withdraw, protecting them against unfavorable conditions such as predation, desiccation, and deposition of particles [1–3]. The operculum is unique in Sabellariidae. It is seen as their fundamental apomorphy [3] and is regarded to be their most important protective and sensory structure [2, 4, 5, 7, 8]. The operculum comprises two lobes, and anteriorly bears one to three rows of strong protective chaetae, the paleae, immediately followed by a row of opercular papillae [2, 4]. Laterally on the lobes, usually, numerous oral or tentacular filaments arise, and a pair of palps completes the anterior appendages. At the dorsal junction of the opercular lobes, a crest is located running from the upper lip towards the opercular crown, called the median ridge, which anteriorly may hold the median organ in certain species [5]. The median organ (as the dorsal hump in larvae) is considered to play a significant sensory role during settlement, metamorphosis, and in adults [5, 6, 9]. This part of the operculum bears eyes in addition to the cerebral eyes in many species [2, 5, 9]. Very likely, these eyes are essential for the escape or shadow reflex when the animals withdraw into their tubes [6].

Although the external morphology of the group is well known, to date, Sabellariidae have seldom been studied by transmission electron microscopy (TEM). These previous investigations include general observations on larvae and metamorphosis [10, 11], oogenesis and spermatogenesis [12, 13] as well as some more preliminary data on the tentacular filaments [14]. Among their sensory structures, only the palps (termed tentacles) have been studied thus far in late larvae of *Phragmatopoma californica* [15, 16]. Recent investigations on the sensory organs in Sabellaraiidae focused on the thus far underestimated importance of the median organ [2, 5, 6, 9], neglecting, however, the possible sensory role of the other appendages.

In Sabellariidae, data on the branchiae are only available on the light microscopic level [17–19]. However, ultrastructural observations exist for several other polychaete species [20–24]. These reveal a certain degree of variability among the taxa investigated besides certain common features such as external ciliation, a comparatively thin cuticle, and epithelia, blood vessels, and coelomic spaces. In addition to the obvious function of oxygen uptake, involvement in osmoregulation and ion exchange has been proposed in *Diopatra neapolitana* [23], and ammonia excretion has been demonstrated in *Eurythoe complanata* [24]. These data highlighted certain inconsistencies, raised questions, and prompted attempts to classify these appendages. The different classifications have been criticized by Belova and Zhadan [21], who claimed a need for revision.

Since the ultrastructure and morphology of the different appendages were unknown, an ultrastructural investigation was designed for *Sabellaria alveolata,* a species common in European waters [5]. In this species, individuals possess all kinds of appendages mentioned. The present study combines light microscopy, scanning, and transmission electron microscopy to describe the structure of these organs. It is supplemented with a few confocal laser scanning microscopy observations using antibodies for neuronal markers to obtain information on the innervation of these structures. The data obtained are used to analyze their functional morphology, to bridge the data gap, and to provide data for future phylogenetic and evolutionary analyses. Finally a revised classification of polychaete branchiae is suggested.

## Results

### Position of appendages

**Fig. 1:**
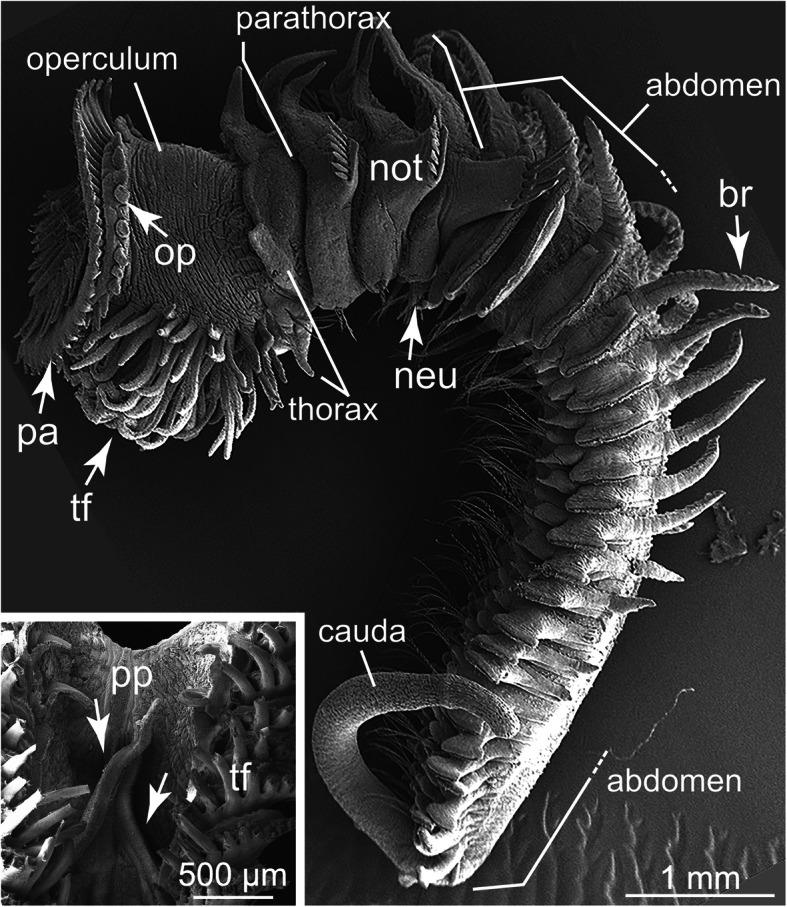
*S. alveolata* from Saint-Efflam, France. SEM. Body regions and diversity of appendages, whole specimen, lateral view. Ring of opercular papillae (op) directly behind the opercular paleae (pa). Ventral side of operculum with numerous tentacular filaments (tf) protruding from the body and surrounding the mouth; first thoracic segment more or less fused with operculum, 2nd thoracic segment bears 1^st^pair of branchiae; three parathoracic segments with capillary chaetae-bearing notopodia (not) followed by abdomen with paddle-shaped notopodia; abdominal neuropodia (neu) with simple capillary chaetae. Branchiae (br) present dorsally on the anterior segments except for the first thorax segment; last branchiae on 14th abdominal chaetiger. The posterior end forms the unsegmentated cauda (cauda). **Inset:** Ventral view of opened operculum. Tentacular filaments partly removed to show pair of palps (pp) and protruding anteriorly. *Abbreviations: br - branchia, neu - neuropodium, not - notopodium, op - opercular papilla, pa - opercular palea, pp - palp, tf - tentacular filament*

The body of *S. alveolata* comprises five distinct body regions: opercular, thoracic, parathoracic, abdominal, and caudal region (Fig. 1). The operculum represents the fused anterior end, the thorax comprises two, the parathorax three and the abdomen a variable number of segments. The different parts are primarily to be distinguished by their different parapodia and chaetae (for details and discussion see [3–7]). The tube-like cauda appears to be unsegmented and is curved anteriorly along the ventral side. Except for the cauda, each of these regions exhibits multiple specialized appendages.

**Fig. 2:**
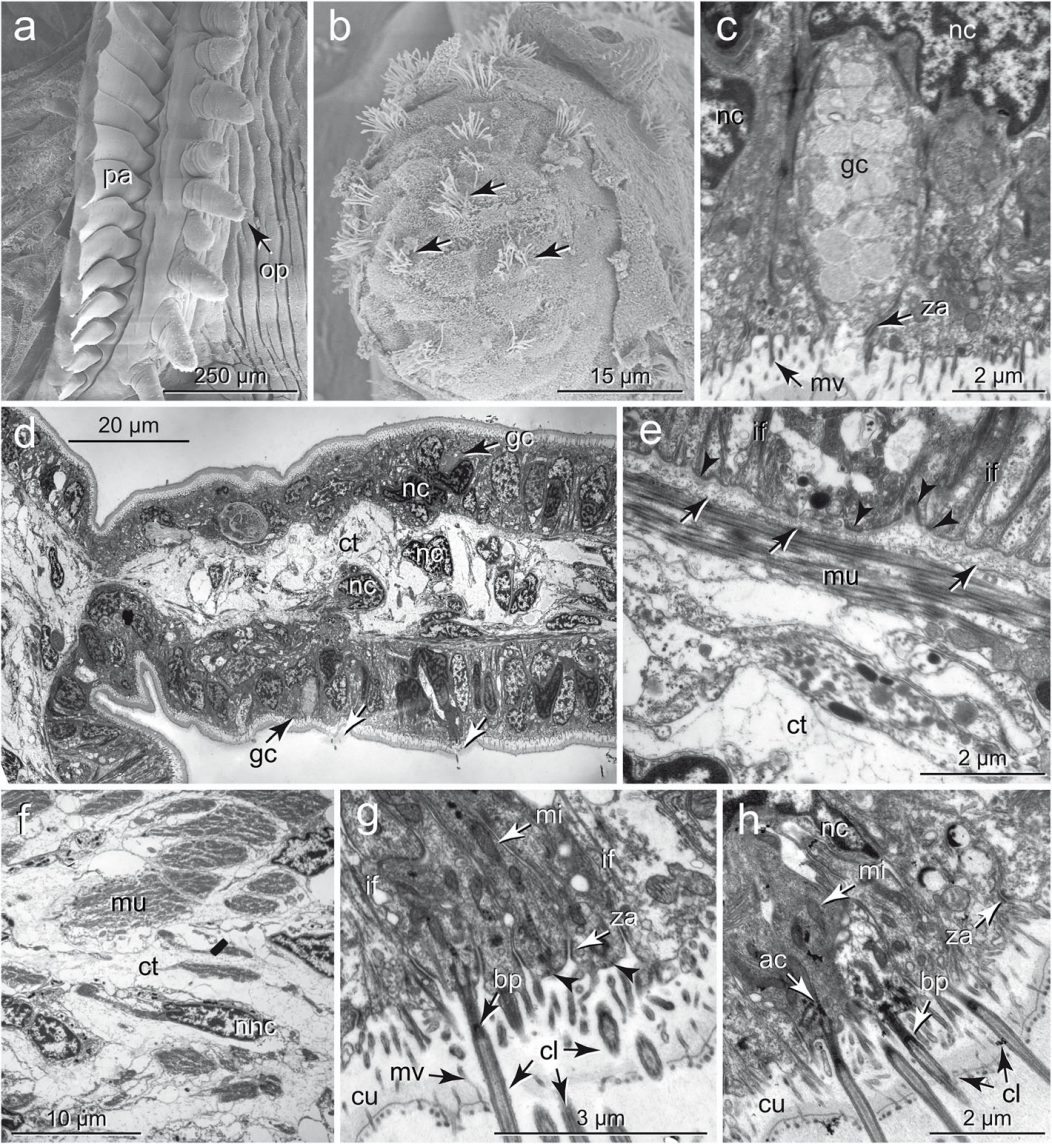
*S. alveolata.* Opercular papillae. **a, b** SEMs. **c-h** longitudinal TEM sections. **a** Finger-shaped opercular papillae (op) located underneath the opercular paleae (pa), circle of papillae surrounding entire head region, **b** Tip of opercular papilla showing several bundles of sensory cilia (arrows), **c** Glandular cells (gc) with electron-lucent content open through indistinct pores in the cuticle, connected to neighboring cells via zonula adherens (za), **d** Longitudinal section of a papilla with prominent epidermal layer surrounding strand of connective tissue (ct) extending from opercular connective tissue. Several glandular cells (gc) between epidermal supportive cells, arrows point to ciliated receptor cells; anterior to the left, figures **c, g, h** represent magnifications from ventral side of (**d**, **e)** ECM between epidermis and connective tissue (arrows). Longitudinal muscle fibers (mu) between connective tissue and ECM; arrowheads: hemidesmosomes, (**f)** Bundles of additional circular muscle fibers (mu) in the connective tissue (ct), (**g, h)** Details of receptor cells with cilia and accessory centriole (ac), microvilli (mv) penetrating the cuticle (cu), groups of receptor cells comprise monociliary and multiciliary cells, cuticle devoid of collagen fibers, receptor cells adjoined to each other and to adjacent supportive cells via zonulae adherentes (za). *Abbreviations: ac - accessory centriole, bp - basal plate, cl - cilium, ct - connective tissue, cu - cuticle, gc - glandular cell, if - intermediate filaments, mi - mitochondrion, mu - musculature, mv - microvillus, nc - nucleus, op - opercular papilla, pa - opercular palea, za - zonula adherens*

**Fig. 3:**
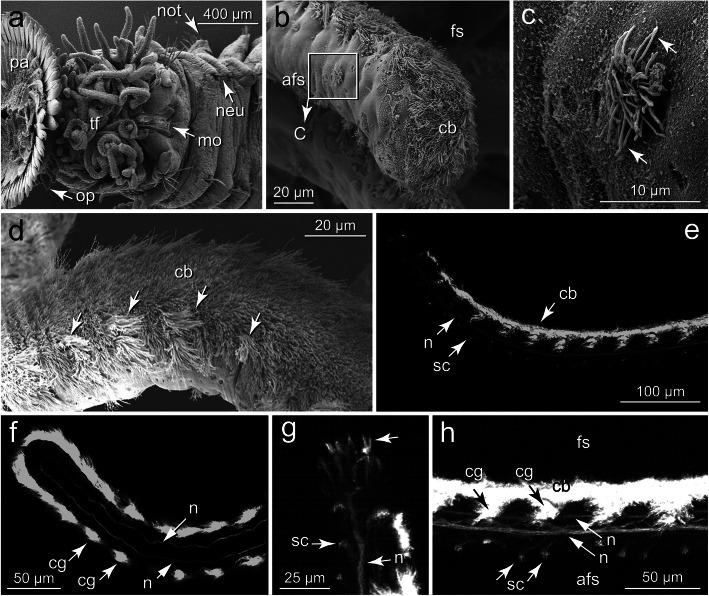
*S. alveolata.* Tentacular filaments. **a-d** SEMs, **e-h** Anti-acetylated α-tubulin-like immuno-reactivity, cLSM z-stacks, **a** Tentacular filaments (tf) situated ventrally on the operculum, **b** Tentacular filament carrying a strong ciliary band (cb) on the frontal side (fs) and a few groups of cilia (boxed) on the abfrontal side (afs), **c** Enlargement of boxed ciliary group from **b** presumably belonging to receptor cells, **d** Tentacular filament with short transverse rows of long cilia (arrows) regularly arranged on each side of the frontal ciliary band (cb), **e** Lateral view of filament, strong ciliary band (cb) and groups of long cilia, tufts of sensory cilia (sc) on the abfrontal side, **f** Two nerves (n) running along abfrontal side of tentacular filament, frontal view, selected z-stack projection to eliminate the strong signal of the ciliary band, **g** Tip of filament with groups of sensory cilia on the very top (arrow), abfrontal nerves (n) branch and their processes giving rise to sensory cilia, **h** Enlargement of the middle part of a tentacular filament, lateral view, each sensory ciliary bundle (sc) connected via a single neurite to the ventrally running nerves, from the same nerve neurites approach transverse groups of long cilia. *Abbreviations: afs - abfrontal side, cb - ciliary band, cg - group of long cilia, fs - frontal side, mo - mouth, n - nerve, neu - neuropodium, not - notopodium, op - opercular papilla, pa - opercular palaea, sc - sensory cilium, tf - tentacular filament*

The opercular region on the anterior end bears three different kinds of appendages. Most anteriorly, it carries a ring of opercular papillae present directly underneath the two circles of opercular paleae. Each papilla is a short, finger-like outgrowth of the anterior body wall and does not surpass the roundish disc of opercular paleae when viewed anteriorly (Figs. 1, 2a). In addition, numerous tentacular filaments insert on the ventral side of the opercular lobes, and the latter fuse on the dorsal side. Several parallel rows of these contractile tentacle filaments are present on each lobe, while each row consists of numerous heavily ciliated tentacle filaments (Figs. 1, 3a). Inside the opercular cavity, formed by the dorsal partially fused opercular lobes, two palps are located and protrude anteriorly (Fig. 1, inset).

Besides the parapodia, branchiae occur in the following thoracic, parathoracic, and abdominal regions. These are paired, ciliated structures present on the dorsal side from the second thoracic chaetiger onward and the posteriorly following segments, diminishing in size continuously (Fig. 1, Fig. 7 A, Fig. 8 A-C). The branchiae are finger-like outgrowths of the body wall emerging just above the notopodia and are absent in the more posterior segments. The number of abdominal segments depended on the size of the animals and was between 24 and 34 in the specimens investigated (*n* = 6), as was the number of branchiae. Approximately 10 abdominal segments were devoid of branchiae in the specimens investigated.

### Opercular papillae

The opercular papillae of *S. alveolata* are located circularly around the anterior operculum, underneath the opercular paleae (Figs. 1, 2a). Each papilla is a single finger-like outgrowth, becoming thinner distally and roundish on the tip, presenting several groups of cilia (Fig. 2b). From their base to the tip, they measured 120–130 μm, and the diameter of the structure was 50–60 μm at the base (*n* = 10). The number of opercular papillae was counted between 24 and 40 papillae (*n* = 6) in our material comprising not only adult individuals. Each papilla consists of epidermis and a central cylinder of connective tissue (Fig. 2d).

The inner, central part of each opercular papilla consists of a 17–23 μm thick cylindrical connective tissue layer, followed by a distinctly separated extracellular matrix (ECM) and the approximately 15 μm-thick epidermis (n = 10). The connective tissue of each papilla is continuous with the connective tissue inside the operculum (Fig. 2d). The connective tissue consists of a loose and irregular network of fine fibrillar material and houses numerous cell bodies of mesodermal cells and muscle fibers. The myofilaments primarily follow the longitudinal axis of the papillae. In the muscle cells, nuclei and mitochondria are located towards the inner side, whereas the myofilaments are close to the ECM. Blood vessels are absent in the papillae (Fig. 2d, e).

The epidermis consists of only a few cell types comprising supportive cells, glandular cells, and sensory cells (Fig. 2d). All cells are interconnected by typical junctional complexes comprising a zonula adherens (Fig. 2c, g, h) followed by a septate junction (not shown). It is covered by an approximately 1 μm-thick cuticle penetrated by numerous microvilli and lacking collagen fibers. The cuticle is composed of scarce fine fibrils which become denser apically, and collagen fibers are absent. It is covered by a thin, sheet-like epicuticle (Fig. 2d, h). The microvilli branch, and surpass the cuticle proper for approximately 100 nm and form an apical layer of densely arranged microvillar tips (Fig. 2G, H). The supportive cells are more or less columnar and are characterized by a prominent basal-apical system of intermediate filaments (Fig. 2E). These run from basal to apical hemidesmosomes located at the bases of the microvilli and thereby connecting the ECM with the body surface. A few glandular cells are interposed between the supportive cells (Fig. 2C, D). They possess electron-lucent, heterogenous secretory content. Apically, a circle of microvilli forms an opening aperture, but the cuticle is not interrupted in these areas (Fig. 2C).

The groups of non-motile cilia present on the surface of the papillae belong to sensory cells without exception. These ciliary tufts are arranged in rows following the longitudinal axis of the papillae (Fig. 2B). Motile cilia are absent on the papillae. Each group of cilia is formed by a few sensory cells, the majority of which are monociliated (Fig. 2G, H). Two types of receptor cells may be distinguished by their different electron-density. The cytoplasm in the more frequent type of these cells is more electron-dense and contains basally located nuclei and numerous oval-shaped mitochondria (Fig. 2D, G, H). These cells are monociliated. Although apical microvilli surround each cilium, these do not form a typical collar around the cilia. A strong bundle of intermediate filaments enters each of the branched microvilli. Basally a small rootlet is attached to the basal bodies of the cilia (Fig. 2G), and an accessory centriole may also be present (Fig. 2H). The other receptor cell type possesses a more electron-lucent cytoplasm (Fig. 2G, H). All receptor cell dendrites contain numerous microtubules following their longitudinal axis.

### Tentacular filaments

**Fig. 4:**
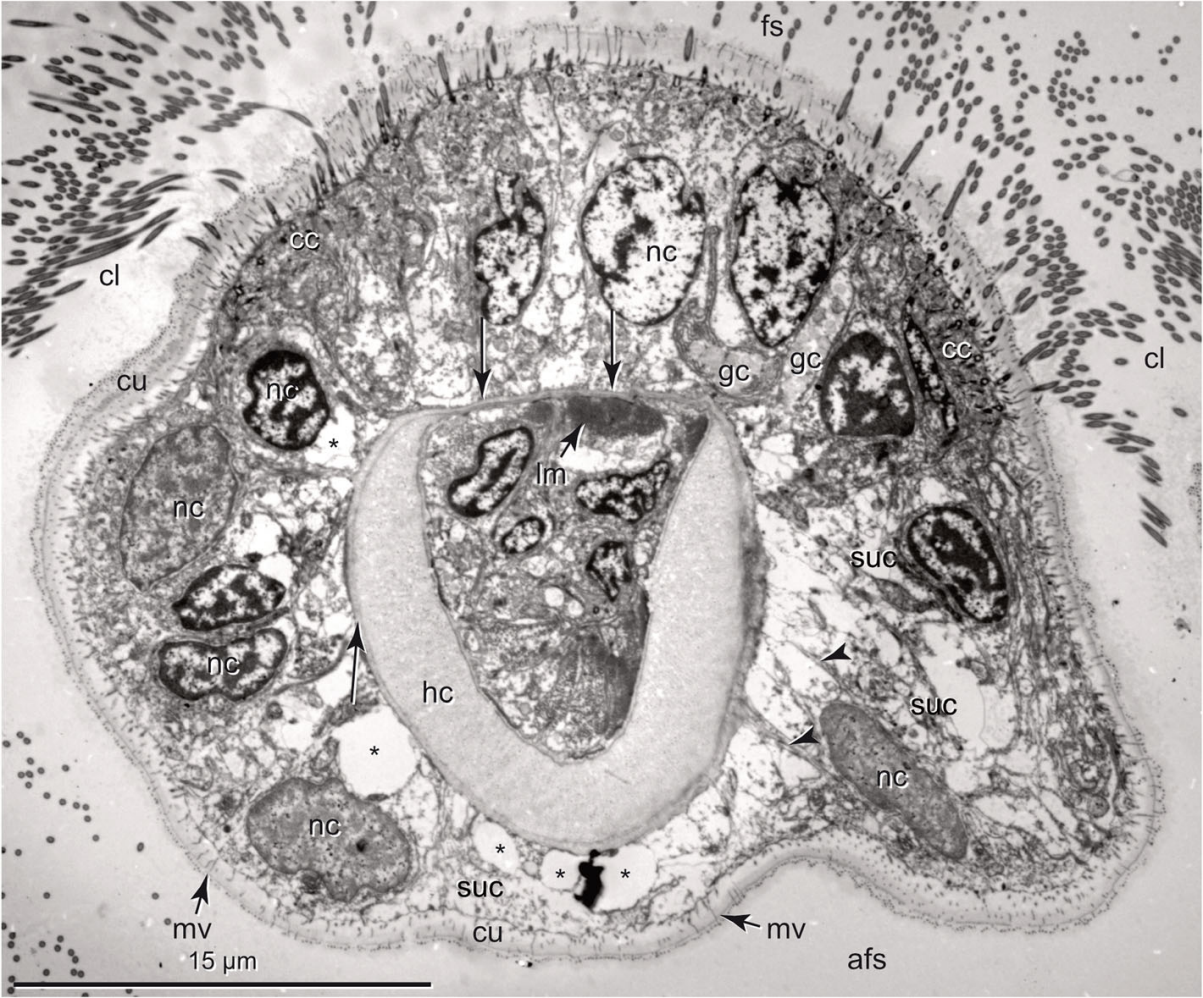
*S. alveolata*. Tentacular filament, TEM, cross-section. Epidermis composed of unciliated supportive cells (suc) with basal-apical intermediate filaments (arrowheads), ciliated cells (cc) and glandular cells (gc). On the frontal side, numerous cilia (cl) penetrate the cuticle (cu) forming the ciliary band. Internally prominent u-shaped, hyaline cartilage structure (hc) located somewhat eccentrically, cartilage structure sheathed by conspicuous ECM, also connecting its upper ends (arrows) and surrounding a central muscle bundle; musculature primarily comprising longitudinal muscle fibers (lm) attached spirally to the ECM and running from the base to the tip of the filament. Glandular cells (gc) interspersed between ciliated cells on frontal side (fs); asterisks refer to intercellular spaces, nuclei (nc) of supportive cells show different staining. *Abbreviations: cc - ciliated cell, cl - cilium, cu - cuticle, fs - frontal side, gc - glandular cell, hc - hyaline cartilage structure, lm - longitudinal muscle, mv - microvillus, suc - supportive cell*

**Fig. 5:**
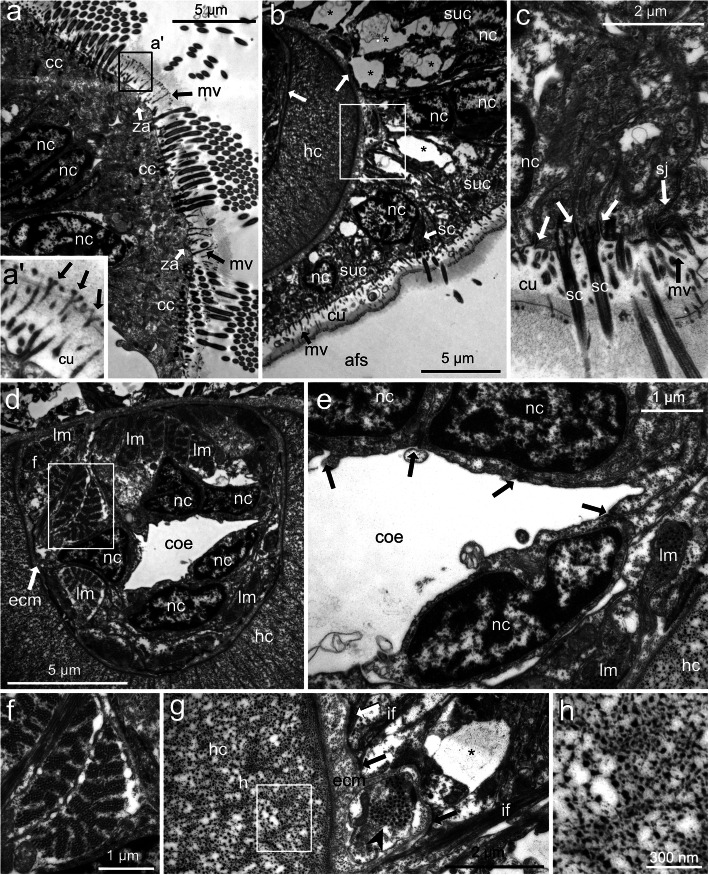
*S. alveolata*. Tentacular filaments, TEM, cross-sections. **a** Three multiciliated cells with long and densely arranged (cc) located on each side of the frontal ciliary band, groups of cilia distinctly separated from each other by the unciliated periphery of ciliated cells, cuticle (cu) devoid of collagen fibers, penetrated by microvilli (mv), note distal branching of microvilli (mv) (boxed and inset **a’**), **b** Right part of u-shaped hyaline cartilage structure (hc) surrounded by ECM and unciliated supportive cells (suc); groups of sensory cilia (sc) located on abfrontal side of filament; boxed area enlarged in (**g, c)** Three receptor cells on abfrontal side, sensory cilia with basal body and short rootlet; cells adjoined by zonulae adherentes (arrows) and septate junctions (sj), **d** Central strand of tissue laterally located in concave part of u-shaped cartilaginous (hyaline) structure; tissue primarily comprises longitudinal muscle fibers (lm) with a few supplementary transverse fibers around a coelomic cavity (coe), **e** Apices of coelothelial cells forming coelomic cavity (coe), cells connected by zonulae adherentes (arrows), **f** Detail of longitudinal and transverse muscle fibers, boxed area of (**d**, **g)** Enlargement of an outer section from b (boxed) showing u-shaped structure surrounded by ECM (ecm), in contact with intermediate filaments (if) via hemidesmosomes (arrows), note small longitudinal muscle fiber outside central strand of tissue (arrowhead), asterisks: intercellular spaces basally between supportive cells, **h** Detail (boxed in **g**) of material comprising u-shaped hyaline cartilaginous structure. *Abbreviations: afs - abfrontal side, cc - ciliated cell, coe - coelom, cu - cuticle, ecm - ECM, mv - microvillus, nc - nucleus, sc - sensory cilium, sj - septate junction, za - zonula adherens*

The tentacular or oral filaments of *S. alveolata* insert on the ventral side of the opercular lobes On each side of the operculum they are arranged in 8–10 rows, each comprising 8–10 filaments (*n* = 3) (Figs. 1, 3A). Thus, a single individual of *S. alveolata* may possess between 128 and 200 tentacular filaments. On the frontal side, each filament possesses a band of dense cilia running from the base to the tip of the tentacle, laterally flanked by numerous transverse bands of longer cilia, each consisting of 3 groups of cilia (Fig. 3 B, D, Fig. 5 A). The abfrontal side of the tentacle is lacking a ciliary band but features some smaller groups of cilia (Fig. 3 B, C, Fig. 4). Each tentacle is innervated by two nerves running along the longitudinal axis on the abfrontal side (Fig. 3E-H). Repeatedly they branch off in a regular pattern, and these processes finally give rise to sensory cilia (Fig. 5G, H). However, some of these proceed towards the lateral groups of longer motile cilia (Fig. 5H).

Each tentacle filament is surrounded by an approximately 1 μm-thick cuticle penetrated by microvilli. The microvilli break through the cuticle, branch distally into two or more branches, terminate in knob-like tips, and carry an apical glycocalyx (Fig. 5A). As on the opercular papillae, collagenous fibers are lacking in the cuticle.

The underlying epidermis is thicker on the frontal side than on the abfrontal side. It is composed of unciliated supportive cells with prominent bundles of intermediate filaments, ciliated supportive cells, and a few glandular cells (Fig. 4). Ciliated and glandular cells have only been found on the frontal side of the filaments. A characteristic feature of the epidermis is the occurrence of comparatively large intercellular spaces, especially between the unciliated cells on the abfrontal side (Fig. 4, asterisks). The frontal ciliary band is formed by these ciliated cells with a few unciliated cells interspersed between them (Fig. 4). The transverse bands of longer cilia, which are present laterally within the longitudinal ciliary band, consist of three prominent groups of cilia. Each group arises form a single cell, each bearing more than 100 cilia (Fig. 5A). The ciliary bundles are between 4 and 8 μm wide. The distance between following ciliary bands is 25–50 μm. Ultrathin sections through these cells reveal that the density of cilia varies between the cells with longer and shorter cilia being denser in the cells with the long cilia (~ 4.25 cilia/μm^2^ vs. 1 cilium/μm^2^). Smaller groups of ciliated processes of receptor cells are poorly distributed on all sides of the tentacle (Fig. 5B, C). They are also visible after staining with antibodies against acetylated α-tubulin (Fig. 3E-H). The receptor cells show the same characteristics as on the opercular papillae (Fig. 5B, C). All epithelial cells are interconnected to their neighbors by apical zonulae adherentes and septate junctions (Fig. 5C).

A prominent, u-shaped skeletal structure observed in cross-sections is present within the epidermis of each tentacle, being its most characteristic feature (Fig. 4). Basally these cartilage-like structures are blind-ending and are not interconnected between the filaments. Extending from the base to the tip, this cartilage-like structure thus forms a trench with its lumen facing the frontal side in each tentacular filament. The cartilage is surrounded by an ECM on all sides, which also connects both upper ends of the trench and thus separates a cylindrical inner strand of tissue from the epidermis (Figs. 4, 5D, G). This strand is continuous with the mesodermal tissue of the body. It consists of connective tissue cells, longitudinal, oblique, and circular orientated muscle fibers comprising the main musculature of the filaments (Fig. 5D, F). Within this musculature, longitudinal fibers strongly predominate. Frequently a central coelomic space was encountered (Fig. 5D). Adhering junctions have been observed between the muscle fibers surrounding the coelomic cavity (Fig. 5E). Besides the main muscle bundle, a few additional but rather thin longitudinal muscle fibers are distributed outside this central cylinder and occur laterally and on the abfrontal side (Fig. 5G). These additional fibers are entirely enclosed by ECM and thus are not epitheliomuscular. On the abfrontal and lateral sides, the cartilage structure is in contact with extensions of the epidermal supportive cells (Fig. 5B, G). This cartilage-like structure appears homogenous in semithin sections and stains red in toluidine blue staining (see Fig. 3 in [6]), whereas in ultrathin sections, it shows a fine fibrillary structure (Fig. 5B, D, G, H). At higher magnification thinner and thicker structures can be distinguished, the larger ones measuring approximately 40 nm in diameter and the thinner ones, half this size (Fig. 5H). In a more or less regular pattern, a smaller number of additional fibers traverse the cartilage structure radially (Fig. 5B, D). Blood vessels were not observed in the tentacular filaments.

### Palps

In the resting position, the two palps of adult *S. alveolata* lie inside the opercular cavity; they are inserted dorsal and lateral to the mouth opening (Fig. 1 inset). On the frontal side, a prominent ciliary band runs from the base to the tip of the palp and is laterally flanked by additional groups of long and densely arranged cilia, which occur in a regular repeated pattern along the longitudinal axis of the palp.

**Fig. 6:**
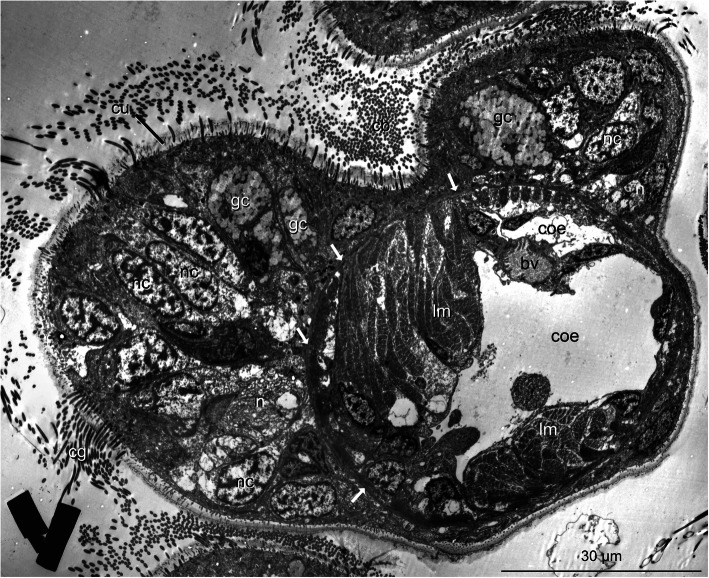
*S. alveolata*. Palp. TEM, cross-section. Epidermis mainly formed by unciliated supportive cell (suc) and two types of ciliated cells: in the median transverse rows of a few cells forming ciliary band (cb) and groove laterally flanked by separate ciliated cells (cc) with denser and longer cilia, ciliary band extends for entire length of palp, glandular cells (gc) with electron-lucent content embedded in epidermis, as well as two nerves (n), ciliated epidermal cells much thicker than unciliated supportive cell opposite to ciliary band, palp supplied with palp canal comprising two tubular coelomic cavities (coe), a single blind-ending blood vessel (bv) and muscular peritoneum; peritoneum primarily comprises musculature compressing the cavity, musculature with thin outer ring of circular muscle fibers (arrows) followed by prominent longitudinal fibers (lm),. blood vessel (bv) runs from the base to the tip of the palp, formed within the strand of ECM lined by peritoneal cells splitting the coelom into two parts, ECM connected to the circular ECM separating mesodermal from ectodermal tissues. *Abbreviations: bv - blood vessel, cb - ciliary band, cc - ciliated cell, coe - coelom, cu - cuticle, gc - glandular cell, lm - longitudinal musculature, n - nerve, nc - nucleus*

The palps are hollow appendages equipped with an eccentrically located cylindrical strand of mesodermal tissue supplied with two closely apposed coelomic cavities (Fig. 6). The following tissue layers can be distinguished: epidermis with cuticle and a peritoneum mainly formed by muscle fibers and a few coelothelial cells separated by a prominent ECM.

The epidermis comprises ciliated and unciliated supportive cells, receptor cells, and glandular cells interconnected by typical junctional complexes (Fig. 6). The epidermis is much thicker on the frontal side than on the abfrontal side. On the frontal side, thinner ciliated cells form a distinct groove. All epidermal cells and the cuticle exhibit an ultrastructure similar to that on the other appendages. Ciliated supportive cells are restricted to the frontal side. As on the tentacular filaments the pattern of cilia is similar: shorter and less dense arranged cilia form most part of the band and groove accompanied by lateral transversely oriented short bands of longer and denser cilia. Each of the groups of dense and long cilia belongs to a single cell (approximately 3–4 cilia/μm^2^ and 100 cilia/cell; *n* = 8). The glandular cells with electron-lucent content open via pores in the frontal cuticle between the less dense ciliated cells. The cell bodies of the glandular cells are embedded between those of the supportive cells in the thicker region lateral to the ciliated groove. Numerous receptor cells are distributed between the supportive cells on all sides. In addition, two prominent nerves run laterally within the epidermis along the longitudinal axis of the palp (Fig. 6).

The coelomic cavities are unequal in size; a smaller lateral cavity is separated from the considerably larger median cavity. Their peritoneum mainly comprises musculature compressing the lumen of the cavity. The musculature consists of an outer ring of thinner muscle fibers with circularly orientated myofilaments followed by inner fibers with longitudinally orientated filaments. These longitudinal fibers are arranged in three distinct groups of different diameters, the smallest in the lateral cavity and the other two in the large cavity. The two coelomic cavities are separated from each other by a strand of ECM and a double layer of peritoneal cells. The strand of ECM is connected to the circular ECM surrounding the entire mesodermal tissues. Inside this sheet of ECM separating the two coelomic spaces, a single blind-ending blood vessel is supplying the palp.

### Branchiae

**Fig. 7:**
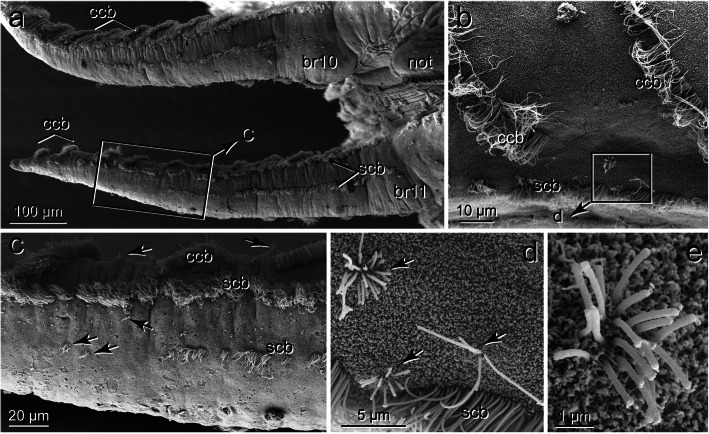
*S. alveolata*. Branchiae, SEM images. **a** Two mid-body branchiae from abdomen (10th, 11th branchia; br10, br11), anterior up, dorsal to the left, each branchia with specific pattern of cilia: two straight and somewhat discontinuous bands (scb) situated on the outward facing side following the longitudinal axis of branchia, anterior row of cilia giving rise to semicircular curved bands (ccb) facing the medial side of branchia, boxed area enlarged in (**c, b)** View from medial side showing curved ciliary bands (ccb) and straight band (scb) with groups of receptor cell cilia in between (boxed and enlarged in (**d**), **c** End of outer straight ciliary band (scb), arrows point to various tufts of receptor cell cilia, **d** Enlargement of the three groups of sensory cell cilia from **b**, rough surface is represented by tips of epidermal microvilli, **e** Single tuft of sensory cilia at high magnification, tips of microvilli create “rough” appearance of surface. *Abbreviations: br - branchia, ccb - curved ciliary band, scb - straight ciliary band, not - notopodium*

**Fig. 8:**
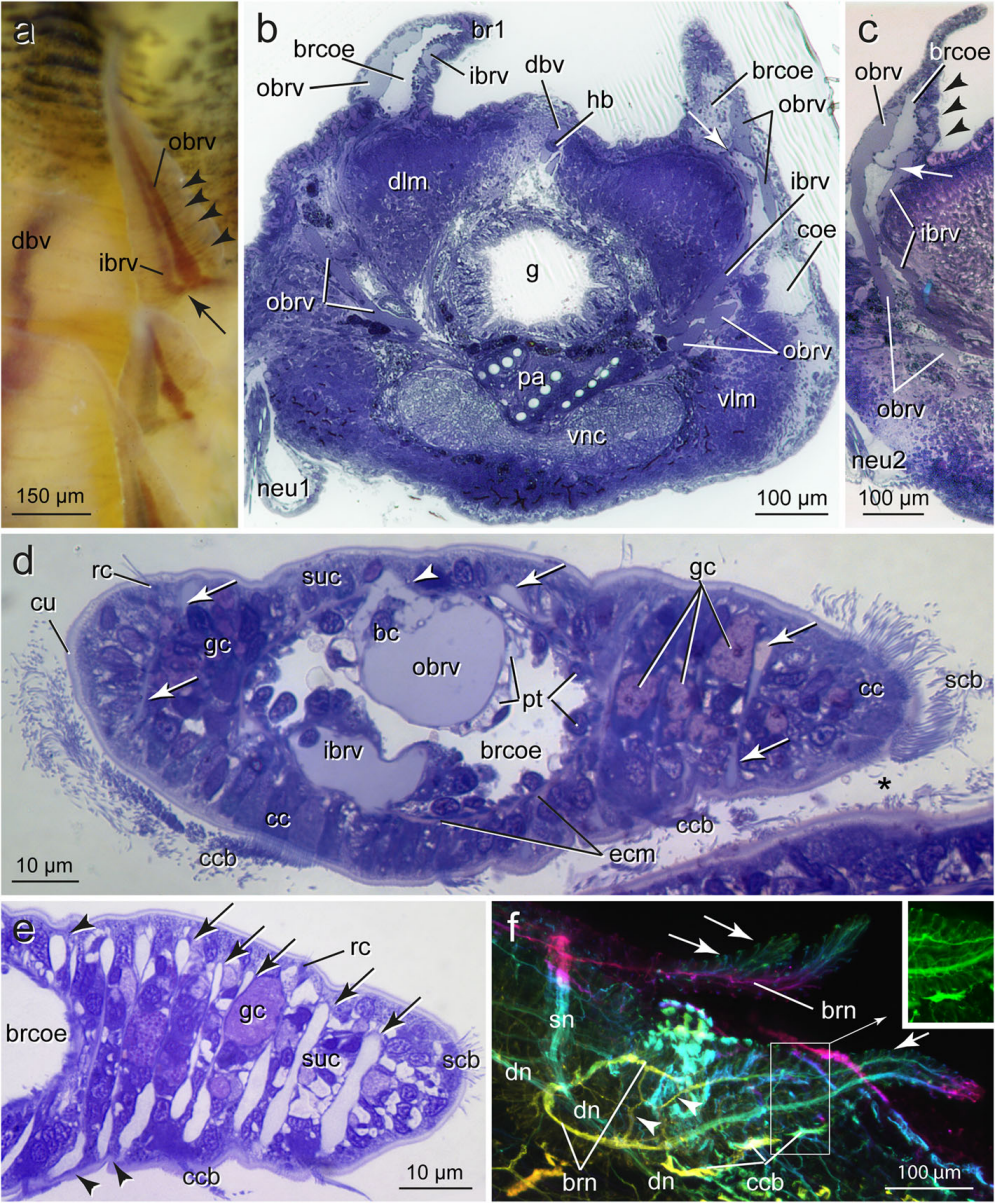
*S. alveolata*. Branchiae. **a** Anterior-most right branchiae with internal longitudinal blood vessels (inner, ibrv, outer branchial vessel, obrv), arrowheads: ladder-like arranged transverse blood spaces, arrow: basal connecting vessel between ibrv and obrv, **b** Branchiae (1st pair) supplied with coelomic space (brcoe), outer and inner vessel (obrv, ibrv), interconnected at base of branchia (arrow), extending beside gut (g), **c** Blood supply of 2nd branchia, arrow: septum with basal connection of ibrv and obrv, separating branchial coelom (brcoe) from body cavity, **d** Right mid-body branchia, flattened side facing dorsum (asterisk), epidermis comprises glandular (gc), receptor (rc), unciliated supportive (suc) and ciliated cells (cc) belonging to straight (scb) and curved ciliary bands (ccb), transverse blood vessels (arrows) between epidermal cells originating from longitudinal vessels (arrowhead); coelom surrounded by peritoneum (pt) and ECM (ecm), **e** Regularly arranged transverse vessels (arrows) between epidermal supportive (suc) and glandular cells (gc), arrowheads: blood spaces close to surface, **f** Innervation of branchiae, anti-acetylated α-tubulin-immune-like reactivity, dorsal view; branchia innervated by two nerves (brn) with common origin where dorsal nerve (dn) meets main segmental nerve (sn), branchial nerves irregularly interconnected (arrowheads) giving off numerous dendritic processes with sensory cilia (arrows and inset). **a** Dissecting microscope, **b-e** LM, semi-thin cross sections; **f** cLSM: depth-coded 0 - 40 μm (yellow to purple). *Abbreviations: bc - blood cell (hemocyte), br - branchia, brcoe - branchial coelom, brn - branchial nerve, cc - ciliated cell, ccb - curved ciliary band, coe - coelom, cu - cuticle, dbv - dorsal blood vessel, dlm - dorsal longitudinal musculature, dn - dorsal nerve, ecm - ECM, g - gut, gc - glandular cell, hb - heart body, ibrv - inner branchial vessel, neu - neuropodium, obrv - outer branchial vessel, pa - opercular palea, pt - peritoneum, rc - receptor cell, sc - sensory cilium, scb - straight ciliary band, sn - segmental nerve, suc - supportive cell, vlm - ventral longitudinal muscle, vnc - ventral nerve cord*

The lanceolate finger-like branchiae of *S. alveolata* of the thoracic and parathoracic segments are somewhat smaller, more slender distally but supplied with a larger base than on the following abdominal segments. The longest branchiae are present on the first abdominal segments (Fig. 1). From there, their size diminishes continuously, and the most posterior ones are just small papillae. Although arising dorsally close to the notopodia from the body wall, they are not part of the parapodia (Figs. 1, 7 A, Fig. 8 A-C). The largest branchiae in the specimens investigated were between 520 and 650 μm long, 180–200 μm wide and 50 μm thick at their bases (*n* = 10). The longer axis was oriented perpendicular to the longitudinal body axis. Anatomically all branchiae exhibited the same structure.

In the resting position, the branchiae are somewhat bent forward and to the dorsal midline, thus pointing with their tips towards the openings of the tubes. The most conspicuous external feature is their characteristic ciliation pattern (Fig. 1, Fig. 7 A-E, Fig. 8 D, E), comprising several bands of densely arranged motile cilia as well as numerous ciliary patches formed by non-motile cilia of receptor cells (Fig. 7 A-E, Fig. 8 D), all of which are arranged in a highly regular and distinct pattern. The motile cilia form two straight longitudinal bands situated on the outer side of each branchia, one of which is located on the outward-facing edge, the other one more anteriorly (Fig. 7A, C). Both bands do not extend to the tip of the branchiae, the latter being somewhat longer. Close to this band, semicircular ciliary bands arise in a more or less regular pattern running on the inner side of the branchiae so that they can hardly be seen if viewed laterally (Figs. 1, 7A-C). These semicircular bands are sickle-shaped with their sagged, concave side oriented towards the dorsum. They are about 50 μm (45–65 μm) apart from each other, thicker in the middle of each semicircle, becoming thinner at their margins (Figs. 1, 7A-C). Accordingly, their number varies with the length of the branchia considered. The longitudinal bands are somewhat discontinuous with some gaps between the groups of cilia (which become larger towards the tips), and finally, the bands end (Fig. 7A, C). Interposed between these cilia are groups of non-motile presumably sensory cilia (Fig. 7B-E). Two groups have been distinguished, one with more numerous and shorter cilia (approximately 10–20 cilia of ~ 1.25–1.5 μm) and another with fewer but longer cilia of different lengths (approximately 5 cilia, 3–6 μm long) (Fig. 7D, E).

In fresh fixed material, the branchiae are yellowish and somehow transparent; thus, their blood vessels are visible under a dissecting microscope (Fig. 8A). Each branchia is supplied by two longitudinal vessels, the efferent and afferent vessels of Meyer [17], here more neutrally called outer (efferent) and inner (afferent) branchial vessel according to their position (Suppl. Fig. S1, Suppl. File 3 Video). These two vessels proceed towards the tip of the branchia in a hairpin-like arrangement with a pointed and extended tip connecting them distally. In contrast to the coelom, which almost reaches the tip of the branchiae, these vessels do not extend into the approximately uppermost quarter of the branchiae. In most preparations, the outer vessel appeared to be thicker than the inner (Fig. 8A-D). Basally at the level of the body surface, both vessels are interconnected by a distinct connecting vessel (Fig. 8A-C; arrows). This point likewise marks where the branchial coelom is separated from the coelom of the trunk by a thin septum (Fig. 8C, Suppl. Fig. S1, Suppl. File 3 Video). The outer and the inner branchial vessels extend deeply into the body, and after separately passing the strong dorsal longitudinal musculature, they turn medially towards the midline (Fig. 8B, C). Their connection to the vascular system of the trunk has not been studied.

**Fig. 9:**
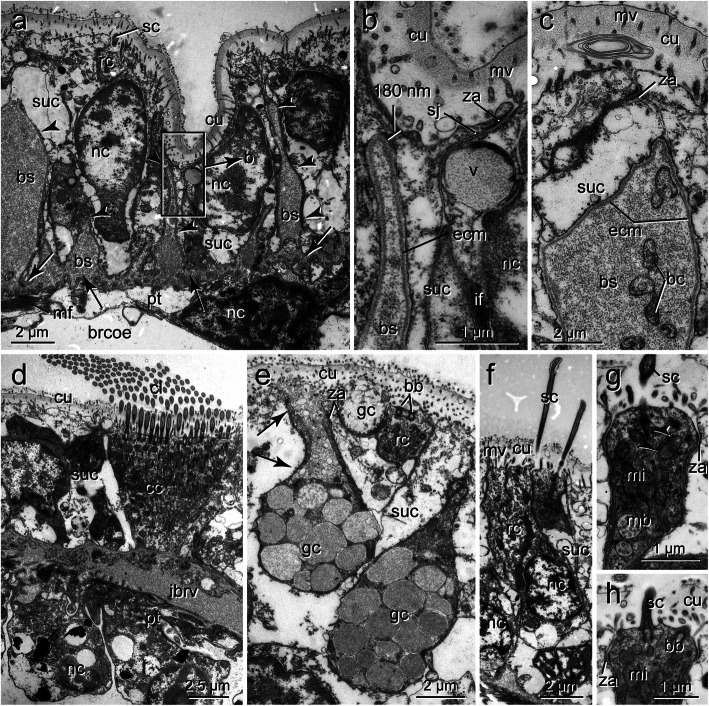
*S. alveolata*. Branchiae, epidermis and peripheral blood vessels, TEM. **a** Unciliated epidermal supportive cells (suc) above branchial coelom (brcoe), basal blood space (bs) gives off several branches (arrowheads) extending towards surface, note receptor cell process (rc) with sensory cilium (sc), **b** Enlargement of boxed area in **a** to show minimal distance between blood space and cuticle (arrow), blood space bordered by ECM (ecm), **c** Blood space (bs) between supportive cells (suc), cuticle (cu) without collagen fibers traversed by a few microvilli (mv), extensions of hemocyte (bc) reach into blood space, **d** Densely ciliated cell (cc) belonging to curved ciliated band between unciliated supportive cells (suc), cells situated above inner branchial vessel (ibrv), coelomic side of vessel bordered by granulocyte-like peritoneal cells (pt) above ECM, **e** Glandular cell necks (gc) and multiciliated receptor cell process (rc) between supportive cells (suc); arrows point to deeply invaginated aperture of type-2 glandular cell, **f** Group of three receptor cells, cut in different planes, two unicilated with cell body (on the left), **g** Dendritic process with diminutive rootlets of sensory cilia (arrowheads), **h** Sensory cilia (sc) and basal bodies (bb) of dendritic process. *Abbreviations: bb - basal body, bc - blood cell (haemocyte), brcoe - branchial coelom, bs - blood space, cc - ciliated cell, cl - cilium, cu - cuticle, ecm - ECM, gc - glandular cell, if - intermediate filaments, ibrv - inner branchial vessel, − mb multivesicular body, mf - myofilaments, mi - mitochondrion, mv - microvillus, nc - nucleus, pt - peritoneum, rc - receptor cell, sc - sensory cilium, sj - septate junction, suc - supportive cell, v - vesicle, za - zonula adherens*

**Fig. 10:**
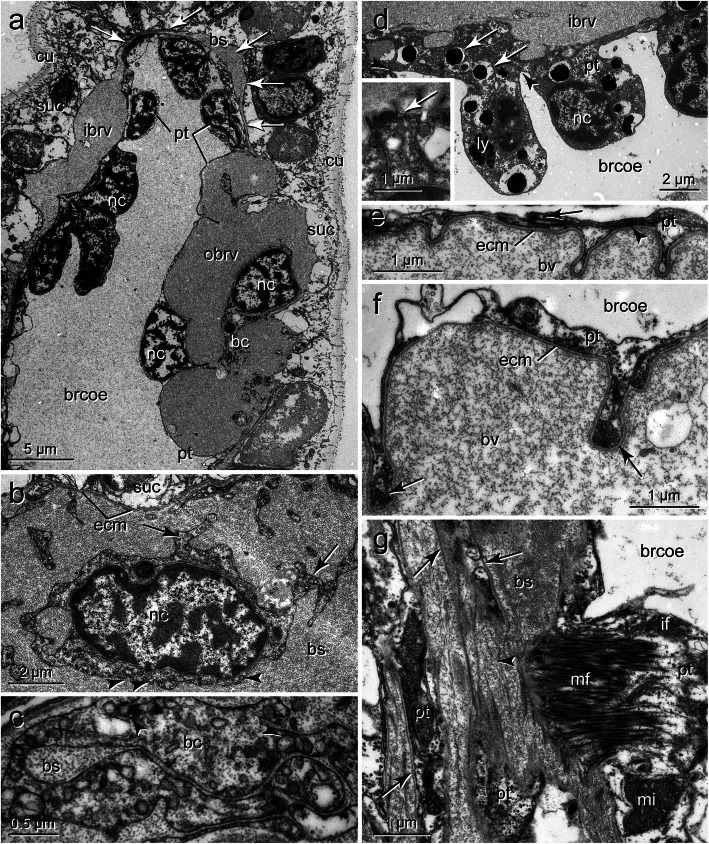
*S. alveolata*. Branchial coelom and blood vessels TEM. **a** Somewhat oblique section through middle region of branchia showing inner and outer branchial vessel (ibrv, obrv) and circular connecting blood space (bs, arrows), note blood cell (hemocyte) (bc) in obrv, peritoneum (pt) formed by cells of varying thickness reaching their maximum thickness around the nuclei (nc), **b** Blood cell with nucleus (nc) and branched sheet-like extensions (arrows), arrowheads point to coated pits, **c** Enlargement of hemocyte process with numerous endosomes, coated vesicles and a few coated pits (arrowheads), **d** Coelomic lining or peritoneum (pt) of inner branchial vessel (ibrv) formed by granulocyte-like cells with numerous electron-dense granules and lysosomes (ly), inset: basal part of granulocyte-like cell facing to blood vessel with bundle of myofilaments (arrow), **e** ECM (ecm) forming wall of blood vessel (bv) covered by extremely thin coelomic lining (pt), arrow points to zonula adherens, arrowhead to septate junction between adjacent peritoneal cells, **f** Coelomic lining (pt) covering blood vessel (bv) composed of myofilament-free and myofilament-containing cells (arrows), **g** Tangential section through several basal regions of blood spaces (bs) and branchial coelom (brcoe); peritoneal cell with myofilaments (mf) and intermediate filaments (if), peritoneal cells followed by ECM (arrows) and blood space (bs), ECM covered by a loose meshwork of collagen-like fibers (arrowhead). *Abbreviations: bc - blood cell (hemocyte), brcoe - branchial coelom, bs - blood space, bv - blood vessel, cu - cuticle, ecm - ECM, ibrv - inner branchial vessel, if - intermediate filaments, ly - lysosome, mf - myofilaments, mi - mitochondrion, nc - nucleus, pt - peritoneum, suc - supportive cell*

Within each branchia, these longitudinal blood vessels are frequently interconnected by transversely oriented ring-like blood vessels (also called blood spaces or blood sinuses) situated in the ECM surrounding the branchial coelom and separating the epidermis from the mesodermal tissue (Fig. 8 D, Fig. 9 A-D, Fig. 10 A, Suppl. Fig. S2). This ECM (about 100 nm thick) also lines the blood spaces in the epidermis (Fig. 9B, C). Thus, the blood vessels represent spaces in the ECM of the epidermis (Fig. 9B, C) or in the ECM between the epidermis and peritoneum (Fig. 9 A, Fig. 10 A-G). In the areas around the longitudinal blood vessels, a network of distinct fibers has been observed just above the ECM in the blood (Fig. 10G).

The ring-like vessels give rise to further transverse blood sinuses extending deep into the branchial epidermis (Fig. 8 E, Fig. 9 A, Fig. 10 A, Suppl. Fig. S2). These blood vessels are arranged more or less leaf-like and give the branchial blood vascular system a ladder-like appearance (Fig. 8A, E, Suppl. Figs. S1, S2, Suppl. File 3 Video). Connections to the longitudinal vessels and their ring-like connecting vessels are difficult to observe (Fig. 8D, arrowhead, 9A, arrows; Suppl. Fig. S2). The number and density (approximately 1 per 5 μm) of the leaf-like blood spaces are more or less constant from proximal to distal. However, the uppermost part of the branchiae is without such blood spaces. When filled with blood, these spaces are up to 5 μm wide but may become rather narrow when they are almost empty (0.4 μm; Fig. 8 D, E, Fig. 9 A-C). When filled, adjacent blood vessels are rather close to each other (Suppl. Fig. S2). In the epidermis, the blood vessels almost reach the apical cell membrane at certain places; mostly, they terminate between 150 and 400 nm below the apical surface (Fig. 9B, C).

Occasionally blood cells hemocytes were found in the blood (Fig. 10A-C, Suppl. Fig. S2). These are characterized by a compact cell body containing the nucleus and irregularly shaped flat processes giving the cells a somewhat dendritic outline. They are not lined by an ECM and thus their membranes border the blood directly (Fig. 10B, C). Numerous coated pits and vesicles, as well as early endosomes, were found in the processes (Fig. 10C). Larger vesicles of the lysosomal system showing different contents were observed in the cell bodies (Suppl. Fig. S2).

Each branchia is innervated by two bundles of nerve fibers originating at the point where the dorsal longitudinal nerve meets the main segmental nerve proceeding from the ventral nerve cord (Fig. 8F). The branchial nerves follow the longitudinal axis of the branchiae and are located on both flattened sides of the coelom in each branchia. They are situated basiepithelially just above the epidermal ECM and are comparatively close to the surface where they cross the intraepithelial blood spaces. They are irregularly interconnected and give rise to numerous dendritic processes with sensory cilia giving the innervation pattern of the branchiae a herringbone-like pattern (Fig. 8F). In addition, some fibers very likely are in contact with the bands of motile cilia (Fig. 8F). The fiber bundles mainly comprise neurites, but a few glial cells were found as well.

As in the other appendages, the epidermis comprises unciliated and ciliated supportive cells, receptor cells, and numerous glandular cells interconnected by typical junctional complexes (Fig. 9A-H, Suppl. Fig. S2). The epidermis is covered by an approximately 1 μm-thick cuticle exhibiting the same structure as described for the opercular papillae (Fig. 9B, C). Unciliated supportive cells are the most abundant cell type. The cells are characterized by a comparatively electron-lucent cytoplasm poor in organelles and nuclei appearing lighter than those of the other cells. The nuclei are located in larger portions of the cells located between the blood spaces (Fig. 9A, Suppl. Fig. S2). In these regions, the epidermis may be approximately 10–15 μm thick. However, above the main blood vessels, the epidermis may be only approximately 2.5–3 μm thick (Suppl. Fig. S2).

Proximally in the branchiae, numerous glandular cells are interposed between the supportive cells. There are 1–2 glandular cells in each fold between two blood spaces. More distally, the number of glandular cells decreases (Suppl. Fig. S2). In contrast to the body wall, only two types of glandular cells were found. The nuclei were situated basally in the cell bodies and contained more heterochromatin than found in the supportive cells. The more frequent type is characterized by an almost electron-lucent secretion which occupies a large part of the cell. The mature secretion appeared to form a coalesced mass with some discontinuous membranes in between. These cells form 2 μm-wide necks reaching the apical level of the epidermis (Fig. 9E). The other glandular cell type is characterized by membrane-bounded secretory granules (~ 1.5 × 2 μm) with granular contents of medium electron density (Fig. 9E). These glandular cells possess elongated necks as well, the uppermost approximately 4 μm form characteristic apertures (Fig. 9E). The junctional complexes are situated at the level of the adjacent epidermal supportive cells. From here the apical membrane of the glandular cell is folded inside resulting in a cylindrical depression of their apical part (Fig. 9E). The cytoplasmic cylinder surrounding this 1.5 μm-wide depression is only 150–350 nm thick. Inside this depression, numerous small vesicle-like particles have been found. In both cell types, circularly arranged microvilli were not observed. Likewise, the cuticle was not modified above the glandular cells. Often a receptor cell process was found close to the glandular cell openings (Fig. 9E).

The curved ciliary bands are usually formed by rows of cells only one cell wide on their narrow axis (Fig. 9E, Suppl. Fig. S2). The straight bands may comprise more cells (Fig. 8 D, E, Fig. 9 E, Suppl. Fig. S2). Depending on the width of the ciliary band, the cells bear rows of approximately 8 to 16 cilia making up more than 100 cilia per cell. There are about 7 cilia per μm^2^. The cilia are anchored in the cytoplasm by a prominent rootlet system comprising a longer rootlet extending for approximately 3.5–4 μm into the cell and a shorter rootlet parallel to the apical cell membrane (Fig. 9D). The rootlet system continues in the basal-apical filament system. Between the rootlets, numerous mitochondria were found. Generally, the cells are trapezoid in cross-section and are broadly anchored on the ECM (Fig. 9D). The nuclei are typically situated laterally to the cilia (Suppl. Fig. S2). Due to their high content in filamentous structures and mitochondria, these cells appear more electron-dense than the unciliated supportive cells.

The receptor cells are typical bipolar cells with their cell bodies situated between the epidermal supportive cells (Fig. 9F, Suppl. Fig. S2). Usually, they form small groups of a few cells. Multiciliated and uniciliated receptor cells were found, all with cilia penetrating the cuticle (Fig. 9F-H). The multiciliated cells bear approximately 6 cilia. The cilia possess a 9 × 2 + 2 axoneme and are anchored by a basal body extending above the cell surface for approximately half their length (Fig. 9H). To these basal bodies, small indistinct rootlets are attached in the multiciliated cells (Fig. 9G). In the dendritic processes, flat cisternae of agranular endoplasmic reticulum are present close to the cell membrane. In addition, microtubules following the longitudinal axis of the processes, mitochondria, and multivesicular bodies were found (Fig. 9G).

The peritoneum is formed by either rather flat sheet-like cells lining the coelom only 30 nm thick in certain areas without nuclei or myofilaments (Fig. 10F, Suppl. Fig. S2) or by cells with larger cell bodies appearing granulocyte-like (Fig. 10D, Suppl. Fig. S2). All cells form a typical epithelium connected by zonulae adherentes and septate junctions (Fig. 10D, E). The sheet-like cells contain bundles of myofilaments following the main axes of the blood vessels (Fig. 10F, G). In these areas, the cells are much thicker (300–400 nm). Depending on their contraction, the cells may form folds extending into the blood vessels (Fig. 10E). The granulocyte-like cells are only present in the peritoneum above the two main blood vessels. They contain numerous electron-dense vesicles of unknown contents (Fig. 10D). Processes of the flattened peritoneal cells with myofilaments extend between these cells (Fig. 10D, inset).

## Discussion

### General aspects

Due to their specific and highly specialized morphology and biology, Sabellariidae is a well-defined taxon, their monophyly has repeatedly been shown in morphological and molecular analyses (reviewed in [3]). On the other hand, their phylogenetic relationships within Annelida are not well resolved. However, most recent phylogenetic analyses indicate that Sabellariidae and Spionidae are sister groups which form a monophyletic clade with Sabellida. The latter comprising Sabellidae, Serpulidae and Fabriciidae in these analyses [3]. Among the different kind of appendages, two appear to be unique to Sabellariidae, namely the opercular papillae and the tentacular filaments [1–4]. However, these structures may represent modified appendages present in other annelids. Palps and branchiae are widespread among polychaetes [4]. Whereas homology of palps is generally assumed and beyond discussion, this may not apply for branchiae [4, 21, 22]. However, due to their structural diversity and non-uniform presence in the various taxa, there is a certain possibility for homoplasy of these appendages. In contrast, recent ultrastructural studies revealed a few common features within certain annelid groups and some of these characters might turn out to represent common derived structures once a broader basis of data exist [20–24]. These questions will be addressed by a comparison of the appendages and their fine structure within annelids.

All four types of appendages studied in *S. alveolata*, opercular papillae, tentacular filaments, palps and branchiae, are outgrowths of the body wall. They comprise epidermal and mesodermal tissues. Whereas epidermis and cuticle are structurally more uniform, the mesodermal structures differ between the four types of appendages. The mesodermal structures may comprise connective tissue, muscle fibers, and coelomic cavities, as well as blood vessels. However, blood vessels and coelomic cavities were not observed in the opercular papillae, blood vessels are absent in the tentacular filaments, and coelomic cavities and blood vessels are only present in the palps and branchiae.

#### Epidermis and cuticle

In all appendages investigated, the epidermis comprises just a few and similar cell types. These include unciliated and ciliated supportive cells, glandular cells, and several types of receptor cells. However, ciliated supportive cells with motile cilia are only present on the tentacular filaments, palps, and branchiae. Typical for multiciliated cells in annelids, accessory centrioles are lacking, and the basal bodies are equipped with two striated rootlets, a short one parallel to the apical membrane and a long one extending deep into the cell body and anchoring the cilium [25]. The density of cilia has only rarely been determined for annelid epithelia [25–27] but in the appendages of *S. alveolata,* it is similar to that of pharyngeal epithelia of other annelids with reported densities of up to 6.5–8.5 cilia per μm^2^ [28, 29]. In these cells, more than 100 cilia per cell may be present [29]. Such densely arranged cilia have been termed compound cilia. The respective cells have been termed ciliophores, if these cilia are anchored by long rootlets and more than 100 cilia per cell are present [14, 27, 30]. However, there are no ultrastructural differences from other ciliated cells, and thus justification of this separation and term appears to be questionable. Generally, densely ciliated cells are present on epithelia transporting and collecting materials as well as on epithelia generating water currents for ventilating the branchiae or swimming. The functional significance of the varying density was not addressed.

The organization of the epidermis is similar to that of polychaetes in general except for the structure of the cuticle [25, 26]. This includes the presence of glandular cells, which are generally abundant in the annelid epidermis [25]. Due to their different functions, glandular cells and their secretion exhibit a high structural diversity, as can be observed in their apical apertures. Depending on the fine structure of the cuticle, these apertures often comprise single or multiple circles of microvilli leaving a more or less distinct central pore in the cuticle for the release of secretions [25].

In *S. alveolata,* the epidermis is covered by a cuticle, similar to a larval cuticle of other annelids in the absence of layers of parallel collagen fibers. Such layers are usually the most characteristic feature of the cuticle in adult annelids of comparable body size [25, 26, 31]. Other sabellariids studied thus far show a similar pattern of cuticular ultrastructure indicating that this is typical of Sabellariidae [6, 9–11, 14, 15]. In addition to Sabellariidae, other exceptions to this general pattern are polychaetes having meiofaunal body dimensions and, more importantly in this context, several members of the so-called basal radiation [32, 33] such as Oweniidae, Magelonidae, Chaetopteridae, Psammodrilidae and Apistobranchidae which comprise larger often tube-dwelling species as well [25, 34–36].

Likewise, there are no differences in the structure of the cuticle between the different body appendages studied here, the operculum or the trunk in *S. alveolata* [6, 26]. In species possessing a cuticle with collagen fibers, the thickness of the branchial cuticle is considerably less, and collagen fibers are reduced or even absent [21, 22, 37, 38]. Most likely, this is indicative that in Sabellariidae the cuticle does not provide a real barrier for the exchange of molecules with the surrounding environment and there seems to be no functional necessity for a thinner or specialized cuticle in epithelia adapted to gas exchange (see also [6]).

In general, the annelid cuticle is a flexible and soft structure through which many substances may diffuse rather than a tight border as the term cuticle may suggest [25, 31, 39]. Moreover, the cuticle is regularly traversed by more or less densely arranged microvilli, their apices forming a cover above the cuticle proper and are in direct contact with the environment. The weak structure of the cuticle might also be an adaptation to the tubicolous lifestyle of all Sabellariidae, which cannot survive outside their tubes after settlement [3]. Thus, their elaborate and firm tubes may have partly taken over the protective role of the cuticle. Absence of collagen fibers and thus similar cuticular features has also been reported for a few species of tubicolous Sabellidae and Serpulidae [40].

#### Receptor cells

Receptor cells occur everywhere in the epidermis of annelids, but on the various types of appendages, they are usually present in higher numbers [25, 41]. Therefore, appendages rich in receptor cells are generally regarded as sensory organs like the antennae, palps, parapodial, and anal cirri [41–44]. On the body and the appendages, the receptor cells may occur scattered between supportive cells or clustered in small groups of mostly just a few cells. These cells are bipolar sensory cells, and their somata are either located in the epidermis or are situated deeper in the body within the nervous system.

The dendritic processes of the receptor cells are generally ciliated, and accordingly, they are often classified by the number of cilia present and whether or not these cilia penetrate the cuticle [43]. The latter, usually called non-penetrative receptor cells, were not encountered on the appendages investigated in *S. alveolata*. Due to their fine structure, receptor cells may be further subdivided into several subtypes [36, 45]. Structural diversity is generally interpreted as indicative of different sensory stimuli or different sensitivity, although the function of these receptor cells is still insufficiently known and mainly speculative [22, 41].

Among the receptor cell types present on the appendages in *S. alveolata,* uniciliated sensory cells predominate. Usually, the latter are characterized by a dendritic process diminishing apically in diameter to approximately 1 μm or less, and thus they form tufts of densely arranged sensory cilia that cannot be assigned to a certain number of cells if viewed with SEM. Moreover, such cells may intermingle with multiciliated receptor cells possessing a wider cell apex. As a rule, these receptor cells possess microvilli accompanying and surrounding the cilia [41, 43, 44]. One specific receptor cell type of this kind exhibits 8 or 10 strong microvilli forming a regular crown around the single cilium and is usually called a collar receptor [46]. Collar receptors frequently occur in aquatic invertebrates and are the characteristic elements of lateral polychaete organs, representing a typical sensory organ present in many sedentary annelids [36, 41, 43]. Unexpectedly these are obviously absent in the appendages of *S. alveolata*. Compared to purely sensory appendages of other polychaetes, the receptor cell diversity appears to be lower in *S. alveolata.* However, it should be noted that only very few species have been investigated for these features [22, 45, 47–51]. These results are consistent with investigations on the median organ in *S. alveolata* and *Idanthyrsus australensis* (Haswell, 1883), and the palps of larvae in *Phragmatopoma californica* (Fewkes, 1889) and *Phragmatopoma caudata* Krøyer in Mörch, 1863 [as *P. lapidosa* Kinberg, 1866] [6, 9, 15, 16, 19]. However, in the latter two species, only multiciliated receptor cells have been identified, and probable occurrence of uniciliated cells is not mentioned [10, 15, 16]. Thus, the paucity of morphologically distinct ciliated receptor cell types appears to be a general feature of Sabellariidae.

### Opercular papillae

A ring of opercular papillae situated immediately beneath the paleae is generally present in members of Sabellariidae [1, 3]. Usually, these papillae are numerous, differing in number, size, and form according to the species and age of the individual considered [2]. The highest numbers recorded are approximately 20 pairs as observed in our material of *S. alveolata,* and, for instance, up to 18 pairs have been reported in *Lygdamis wambiri* [1]. The number of papillae is age-dependent, and the development of these papillae starts with only one pair in late larvae [9, 52]. In larger specimens, they are often not easy to count, especially if prepared and mounted for SEM. This challenge might be the main reason why so few authors provide numbers in their descriptions as, for instance, are given in Capa et al. [2]. Although these appendages were noted in certain previous studies on the morphology of the anterior end [19, 53], a more precise view of the structure of these appendages was thus far unknown. If viewed with SEM, the opercular papillae with their tufts of sensory cilia distributed all around them (e.g., our Fig. 2B; Fig. 1 in [15]; Fig. 2d, e in [9]) somehow resemble parapodial cirri of other polychaetes [22, 41]. Accordingly, the first pair of these appendages appearing in late pelagic larvae in close proximity to the provisional chaetae has been called cirri or opercular cirri [10, 15, 52, 54]. Somewhat later, after metamorphosis has started, and the provisional chaetae have been replaced, these cirri are situated beneath the first formed paleae and become the first pair of opercular papillae [9, 52].

Generally, the opercular papillae can be classified as outgrowths of the anterior body wall. Due to the presence of connective tissue and musculature, the appendages are moveable structures. In the adult worms, the opercular papillae are situated close to the foremost position of the sabellariid body. Together with the tentacular filaments, the opercular papillae represent those structures which are most likely the first to come in contact with all kinds of potential sensory stimuli when the animals open the operculum and are in the feeding position (see supplementary video in Meyer et al. [6, 55]). The absence of coelom, blood vessels and motile cilia indicate that the papillae are neither involved in feeding nor respiration. Instead, the high number of tufts of sensory cilia likely indicates a predominantly sensory function, irrespective of the fact that the receptor cell diversity is lower as in the appendages of the few errant polychaetes studied for this character [22, 47, 56]. Regarding receptor cell diversity and density, sabellariids notably have a completely different life strategy combined with immobility, presumably requiring a different and specific adapted sensory system [5, 6, 9]. Whereas the median organ is most likely involved in triggering of the shadow reflex [2, 5, 6], mechanical or chemical stimuli may play a significant role in the function of the opercular papillae including triggering the withdrawal of the animal into its tube. Unfortunately, our immunostainings did not allow for clarification of the innervation pattern of these appendages, but we assume that the various nerve tracts innervating the operculum as described and imaged by Orrhage [19] in *S. alveolata* include the efferent fibers of the receptor cells. Out of these, the nerves innervating the lateral parts of the operculum or the outer paleae (nmlo1, nmlo2, and nmoop in [19]) appear to be the best candidates for this function. All nerves innervating the corresponding part of the operculum have been shown to represent parapodial nerves emanating outside the brain from the central nervous system by Orrhage [19].

### Tentacular filaments

Filamentous appendages inserting on the ventral and inner side of the operculum are present in most sabellariid species except for those of *Phalacrostemma* [1, 3]. These branched or unbranched appendages are commonly called tentacular or oral filaments and may occur in high numbers, as is the case in *S. alveolata* [3, 19]. They are involved in the transport of food particles to the mouth and sediment particles for tube construction to the building organ on the ventral side [3, 14, 19, 55, 57, 58]. The particle transport mechanism has been analyzed in high-speed video recordings [14, 55, 58]. In active animals, these appendages are exposed to the exterior and are widely stretched out into the surrounding medium ([55]; see Fig. 1A-D in [5], and supplementary video in [6]). The tentacular filaments are thus the second type of anterior appendage coming in contact with various sensory stimuli and are most likely important sensory structures as well. However, their extensive ciliation, in addition to the sensory cells, speaks in favor of a double function: sensing as well as particle selection and collection.

The external morphology and the histology of the tentacular filaments are comparatively well known [14, 18, 19, 55, 58, 59]. These observations are complemented by preliminary ultrastructural observations of the tentacular filaments by Riisgård and Nielsen [14]. We confirm in our present investigation the general structure of the ciliation pattern of these appendages and clarify some discrepancies. In all studies, frontal cilia have been described as forming a continuous ciliary band, and a more or less distinct groove is formed proximally, which is absent distally [19]. These cilia are supplemented by longer cilia extending laterally from this band. As noted by Riisgård and Nielsen [14], these are made up of three bundles of cilia, each of which belongs to a single cell. So these lateral cilia are formed by three longitudinal rows of densely ciliated cells separated by unciliated cells at regular intervals. These cilia have been called compound cilia, spikes, or grouped frontal and lateral cilia [14, 55, 58]. However, there is no structural evidence that these cilia are somehow structurally interconnected [58].

As in the studies of Dubois et al. [55, 58], we were not able to detect the so-called cirri, somehow separated smaller bundles of long cilia adjacent to the lateral groups of dense cilia, described by Riisgård and Nielsen [14]. It has been argued that they represent artifacts [58]. From live and recorded video observations, it is evident that the lateral grouped cilia often project orthogonally from the surface of the tentacular filaments and appear to be immobile [14, 55, 58]. Nevertheless, according to Riisgård and Nielsen [14], these cilia sporadically bend in the downward longitudinal direction of the tentacular filament. Movability of these cilia has been confirmed, but the direction of ciliary movement has been observed as oblique towards the frontal surface by Dubois et al. [55, 58]. In any case, these previous observations of the activity of these cilia are consistent with our findings on the innervation pattern, strongly suggesting that beating of these long cilia is under nervous control.

Most authors observed that the epidermis is thicker on the frontal side, where the ciliated cells are located [14, 19, 59]. Glandular cells have also only been found on the frontal ciliated side and have been shown to represent only acid mucopolysaccharides belonging to a single type [55]. The conspicuous gutter-like structure situated beneath the epidermis confirms their hyaline cartilaginous nature [14, 19]. However, in contrast to Riisgård and Nielsen [14], it is not part of the basal membrane or ECM, but rather, clearly separated from the ECM and situated beneath it. Likewise, the views that this structure is an artifact and represents an extraordinarily distended basal lamina [58] or represents a blood vessel [18] have to be rejected. Since it is entirely cell-free, it should not be termed connective tissue. The two ends of the “U” on the frontal side are connected by the ECM surrounding the entire element. Here it is underlined only by muscle fibers; the presence of a ligament-like structure as discussed by Orrhage [19] in *S. alveolata* cannot be confirmed or may be represented by the ECM.

The movability of the tentacular filaments has been described in detail [14]. Most likely, this movability is due to their cartilaginous structure in combination with the central musculature enclosed by the cartilaginous structure. In this musculature, longitudinal fibers predominate, and these may be responsible for bending the tip, followed by a spiral contraction of the entire tentacle [14]. Stretching might be a more passive process mainly mediated by the cartilaginous structure serving as a kind of flexible skeletal element. The small additional longitudinal fibers situated on the outer side of the cartilaginous structure are described here for the first time. However, their function (support of the central musculature or their antagonist?) could not be clarified. Observations of the presence or absence of food particles in the surrounding medium are correlated with the motion of the tentacles. Furthermore, their ciliation supports our view that the entire system is under nervous control and that at least in part, the different receptor cells present on these filaments are responsible for the necessary sensory input to trigger the uptake of particles.

We can confirm the presence of a coelomic cavity noted by Orrhage [19]. Typical epithelial junctional complexes are present between the cells bordering the cavity, demonstrating that these spaces in fact represent coelomic cavities sealed by a myoepithelium [60–62]. However, blind-ending blood vessels, as described in the older literature [17–19], are absent in the tentacular filaments. Most likely the cartilaginous structure was mistaken as blood space. Thus a prominent role in gas exchange can be excluded. Whether these appendages are homologous to branchiae or unique for Sabellariidae as discussed by Rouse and Pleijel [4] appears to be answered by the latter hypothesis given their different structure and especially the lack of blood vessels and blood supply.

### Palps

The presence of two so-called peristomal palps in larval and adult members of the Sabellariidae has already been observed [2, 7, 19] and their function in chemo- and mechanoreception as well as in feeding behavior assessed [10, 14, 15, 54, 63]. However, ultrastructural analyses of adult palps are rare in sabellariids and mainly based on light microscopic observations [19, 55] or focus on the larval palps [15].

The present ultrastructural analysis of the palps of adult *S. alveolata* shows strong similarities to the structure of the larval palps of *Phragmatopoma californica* [15]. In brief, the palp is ciliated and grooved on the frontal side along the longitudinal axis and exhibits two coelomic cavities separated by a double strand of peritoneum containing a single blood vessel within its ECM. In the epidermis, two prominent nerves are present. This pattern is somewhat similar to earlier descriptions of sabellariid palps [15, 18, 19, 59] and in particular it is similar to the grooved palps present in Spionida [4, 64, 65].

The palps of *S. alveolata* and other sabellariids are multifunctional organs that might have different tasks regarding the developmental stage of the animal. In larval stages, it is assumed that they have a minor part in feeding but mainly function in mechano- and chemoreception. The prominent ciliary band and additional groups of cilia, in combination with the presence of glandular cells, make them a highly suitable structure for locomotion or transportation [66]. In late larval and juvenile stages, the palps might be responsible for substrate recognition, settlement and the building of the tube, as they sense different chemical signals allowing the animals to recognize others and attach their tube, thus forming the famous large reefs [67, 68].

### Branchiae

#### General features of annelid Branchiae

In annelids, gas exchange may occur through the entire body wall, parapodia, or specialized organs commonly termed branchiae or gills [4, 20, 21, 69]. Polychaete branchiae are generally outgrowths or extensions of the body wall often associated with the parapodia or arising separately from the dorsum [4, 20, 69]. As such, they are composed of epidermis, nerve fibers and receptor cells, blood vessels, musculature, and often coelomic cavities. The occurrence of branchiae is restricted to specific taxa, and especially polychaetes with small body dimensions often don’t possess branchiae. In Clitellata, branchiae are usually absent, and their occurrence is a rare exception [70, 71]. In polychaetes, they are generally found in Amphinomidae, certain Errantia, and many Sedentaria [4, 20, 72]. Accordingly, they exhibit a certain diversity in structure, form, and position, although several common features have been recognized. The highest complexity thus far has been observed in hydrothermal vent annelids and Siboglinidae [38, 45, 73–75].

Thus far, the ultrastructure of polychaete branchiae has been studied in multiple species, not representing close to the entire range of taxa possessing these organs [4, 20–24, 37, 38, 45, 75–83]. Furthermore, recent investigations indicate that our knowledge of several species investigated previously may still be incomplete [21, 22]. Possibly the entire range of diversity may still not be known.

Besides the mentioned diversity, polychaete branchiae show several common features: most polychaete branchiae studied so far are equipped with numerous motile cilia arranged in bands or tufts responsible for generating water currents for an effective, continuous, and powerful exchange of the surrounding water [20]. Thus, the elaboration of concentration gradients above the respiratory surfaces is avoided. In addition, the cuticle is generally thinner above the respiratory epithelia than in other body regions. Polychaete branchiae usually contain a loop of blood vessels giving rise to capillaries basally extending into the epidermal cells [4, 20, 69, 72]. The vessels of this loop are commonly termed afferent and efferent vessels, and within the branchiae, they are differently interconnected. From these vessels, more or less numerous blood spaces or sinuses extend into the epidermis, making the diffusion distances as small as possible. In most cases, the branchiae include mesodermal structures such as muscle fibers and often coelomic spaces. Lastly, branchiae are innervated and bear numerous receptor cells.

#### Sabellaria Branchiae

In *S. alveolata*, the branchiae exhibit the densest ciliation pattern observed, exceeding those of the tentacular filaments and palps. Data on ciliatation density in branchiae are rare; mostly, it is mentioned that a dense ciliation is present [38, 75, 81]. Estimations made based on published micrographs on ciliated cells leads us to assume that the density observed in *S. alveolata* appears to be among the highest described [22]. As in *S. alveolata,* each ciliary band is formed by a single row of ciliated cells in most other polychaetes, such as in the amphimonid *Eurythoe complanata* (Pallas, 1766)*,* the polynoid *Branchipolynoe* sp. and in the ophelliids *Ophelia limacina* (Rathke, 1843)*, Ophelina acuminata* Örsted, 1843 and *Euzonus arcticus* Grube, 1866 [21, 22, 77]. This feature is probably characteristic of alvinellids and trichobranchids as well, although not explicitly mentioned [38, 79]. In contrast, in an undescribed deep-sea orbiniid, the ciliary band is formed by several rows of cells [78]. This can be confirmed by our unpublished data on the intertidal orbiniid *Scoloplos armiger* (Müller, 1776), and thus far, Orbiniidae appears to be the only diverging example. In annelids, only three species have been described thus far possessing branchiae without cilia: *Sternaspis scutata* (Ranzani, 1817), *Travisia forbesii* Johnston, 1840 and *Arenicola marina* (Linnaeus, 1758) [21, 37, 76]. In these species, ventilator currents are solely generated by the peristaltic movements of the body wall [37].

As a rule, the cuticle and epidermis are considerably thinner on branchiae than on other parts of the body [21, 22, 37, 78]. Besides reduction of total thickness, mostly between 1 and 2 μm, the number of layers of parallel collagen fibers forming a net-like structure is reduced, or even lacking [22, 25, 76]. On initial observation, *S. alveolata* does not seem to follow this general pattern, since its cuticle is of similar thickness on all body regions. However, as discussed above in *S. alveolata* and other in sabellariids studied thus far, the entire cuticle is highly reduced.

Except for noting the occurrence of receptor cells and neurite bundles [37, 38, 75, 81, 82], innervation of branchiae has usually not been studied in detail. Receptor cells found on branchiae usually have cilia penetrating the cuticle, and the formation of small groups of uniciliated and multiciliated receptor cells has been described [22, 45]. Multiciliated cells are most common in *Paralvinella hessleri* Desbruyères & Laubier, 1989 (Alvinellidae), whereas in *Eurythoe complanata* (Amphinomidae) uniciliated receptor cells predominate. Our findings for *S. alveolata* most closely resemble the former scenario*.* However, it must be noted that very few species have been studied.

The innervation of the branchiae usually originates from the ventral cord via the segmental nerves [22, 45, 83]. In *Eurythoe complanata*, the branchial nerve branches off from the main segmental nerve, which innervates the parapodium and also comprises efferent fibers from the dorsal cirrus. The situation appears to be similar in *Terebellides* cf. *stroemii* Sars, 1835 (Trichobranchidae), *Cossura pygodactyla* Jones, 1956 (Cossuridae) and *Paralvinella hessleri* (Alvinellidae) [45, 83]. Depending on the structure of the respective branchiae, these nerves may split within the branchiae as in *S. alveolata,* wherein the two nerves mark the extension of the branchial coelomic cavity. In *E. complanata,* the motile cilia are innervated by separate neurite bundles [22], which is not the case in the other species studied, including *S. alveolata*.

In annelids possessing a blood vascular system, the branchiae are supplied by efferent and afferent vessels, which in the branchiae are variously connected and often give rise to blind-ending blood spaces (blood sinus). The latter often extend deep into the basal regions of epidermal supportive cells [21–23, 37, 81]. Efferent and afferent vessels unite distally forming a hairpin-like loop as, for example, observed in *Eurythoe complanata* or *Osedax mucofloris* Glover, Kallstrom, Smith & Dahlgren, 2005 [22, 75]. Occasionally this loop tapers distally and forms a single blind-ending vessel as observed in the present study. Blood is mainly driven back into the body by contraction of the branchial musculature, which is present at least along these longitudinal branchial vessels. The diffusion distances are often calculated to be 1–3 μm [21, 38, 79]. However, in such measurements, generally, the thickness of the cuticle is included, which might not be accurate since, as discussed above, the cuticle in these areas very likely does not form a diffusion barrier. More reliable might be those values including the smallest thickness of only the epidermal cells where the blind-ending blood spaces most closely approach the apical side. In doing so, values of diffusion distances diminish to approximately 130–350 nm [22, 23, 38], within the same range as that observed in *S. alveolata*. The micrographs published suggest that this also applies to other species [21, 38]. Outside such lacunae and sinuses, the thickness of the epidermis is reported to be approximately 5–10 μm, twice that of *S. alveolata*.

The structure of the blood spaces and blood vessels in *S. alveolata* is typical for coelomate invertebrates in general; they are lined by the ECM of the adjacent epithelia, and an endothelium is always absent [37, 60, 72, 84–86]. This ECM is also called vascular lamina and in larger vessels this lamina may be invested with collagen fibers as observed in the larger branchial vessels of *S. alveolata* [60, 72]. The ECM surrounding the afferent and efferent vessels is usually lined by peritoneal and epidermal cells or only by peritoneal cells [21, 22, 37, 38]. The peritoneal cells, or part of them, are usually differentiated as myoepithelial cells, and these cells are responsible for the blood flow inside the branchiae. The same is true for the vessels connecting afferent and efferent vessels, whereas in the terminal branches (blood spaces or sinus) the ECM may only be lined by epidermal cells. As such these blood spaces are non-contractile. In addition, a few peritoneal cells may be podocytes as observed in *Thoracophelia arctica* (Grube, 1866) (as *Euzonus arcticus*) [22]. Since such cells are not very frequent and hard to detect, they may also be present in other species with coelomate branchiae. Hemoglobin is usually extracellular in annelids, and only a few cells are present in the blood. The blood cells or hemocytes present in *S. alveolata* exhibit the same structure and high endocytic activity observed in other species [21, 37, 87].

#### Classification of Branchiae in Annelida

Thus far two attempts have been made to assign the diversity of annelid branchiae to a few different types [81, 88]. Stekol’shchikov [88] and Storch and Alberti [81] distinguished three or four different types of annelid branchiae, respectively. Both classifications appear to be rather different not having much in common. The observations leading to these classifications rely on histological observations and early ultrastructural studies. Since these times more detailed studies have been carried out and more data were collected.

Stekol’shchikov [88] recognized (1) capillary branchiae, (2) semi-coelomic branchiae and (3) coelomic branchiae. Capillary branchiae are characterized by afferent and efferent vessels, intraepidermal vessels or capillaries, and a coelomic cavity. This type is regarded to be widespread in polychaetes. Semi-coelomic branchiae possess one or more blood vessels close to the gill coelomic cavity and are present in Nephtyidae, Sabellidae and Serpulidae. In coelomic branchiae blood vessels are absent altogether (present in Glyceridae and Capitellidae). Whereas type 1 and 3 more or less resemble our types 1 and 3 (see below), the second type mentioned in [88] will not be discussed here, because these structures represent the palps and are not branchiae in Sabellidae and Serpulidae [31, 81, 89, 90]. Unfortunately, no new data are available for Nephtyidae.

Storch and Alberti [81] distinguish (1) branchiae with true blood vessels bordered by epitheliomuscular cells and absence of peripheral vessels, (2) branchiae with partly opened vessels, (3) branchiae with blood spaces directly below the surface epithelium not lined by coelothelial cells, and (4) branchiae with blood spaces within the surface epithelium extending from a central blood space. The first type has been assigned to Spionidae, the second type to Opheliidae and Orbiniidae, the third type to Pectinariidae, Terebelidae and Alvinellidae and the forth type as the most common in polychaetes [81]. The pros and cons of these classifications were discussed by Belova and Zhadan [21], and it was concluded that these classifications are in need for revision. The main weaknesses and flaws based on the latter classification are caused by the fact that it relies on partly incomplete and preliminary observations and a diverging interpretation of the blood vascular system. For instance, in terebellids and alvinellids only the lamellae or filaments were investigated neglecting the stem comprising mesodermal structures including coelom [38, 77, 79] and similar applies for Opheliidae [21]. For similar reasons the observations in *Malacoceros fuliginosus* (Claparède, 1868) [81] may be incomplete as well and a re-investigation of spionid branchiae is desirable. Nowadays the structure and interpretation of the ultrastructure of the blood vascular system as spaces within adjacent epithelia is generally accepted and beyond discussion [60, 72, 84–86]. Based on the data available, we suggest that the annelid branchiae may be divided into three different types.

1. Branchiae with coelom, mesodermal structures and blood vessels

As in *S. alveolata*, a coelomic cavity following the main blood vessels has been observed in many species [21, 37, 38, 76, 79–81]. In contrast to the hydrothermal vent Terebellida, the absence of a coelomic cavity has been reported in the intertidal *Terebella haplochaeta* by Wells et al. [91], but very likely this is due to a misinterpretation of their histological sections (see, e.g., Fig. 12 in [91]). It is unknown whether the coelomic cavities are continuous with the segmental coelom in these species or whether they are separate, as observed in the present study. If the branchiae bear lamellae, filaments, pinnules, and other structures, the coelomic cavity or muscle fibers may reach into these branches [38, 79]. Alternatively these branches simply comprise epidermal structures with extensions of the blood vessels bulging into the ECM of the epidermis [74]. This latter feature has been observed in the branchial leaflets of *Lagis koreni* Malmgren, 1866 [as *Pectinaria koreni*], and most likely, it was erroneously described as a separate type of branchiae by Storch and Alberti [81]. This type is similar to type 1 of Stekol’shchikov [88].

To our present knowledge the type-1 branchiae have only been proven to occur in Sedentaria except Orbiniida (see [32, 33] for taxon composition) which may be indicative for a common origin in the sister group of Orbiniida comprising the remaining Sedentaria. Within this clade branchiae may have been lost repeatedly and loss of external ciliation may have occurred in Arenicolida and Travisiidae. However, there is still a significant lack of data; for instance, branchiae of Scalibregmatidae have not been studied by TEM. These three taxa very likely are closely related and may form a monophyletic group [92].

2. Branchiae with mesodermal tissue and blood vessels but without a coelomic cavity

More rarely, the space between the two main vessels is completely occupied by mesodermal cells, mainly musculature [22, 78]. This type has not been recognized to date and has only been found in Amphinomidae and Orbiniidae. Their evolution cannot be assessed until it is known whether this type is also present in Eunicida. So far only the epithelial cells have been investigated in the latter taxon [23].

3. Coelomic branchiae

In certain species with a reduced or absent blood vascular system, the coelom may take over the function of the blood vascular system [77]. Consequently, branchiae, if present, are of different structure and commonly called coelomic branchiae such as those occurring in glycerids, hydrothermal vent polynoids and capitellids [21, 77]. These species usually appear red due to the presence of hemoglobin in the coelomic fluid or the coelomocytes. This type is identical with the third type in [88]. Due to a reduction of the blood vascular system and their positions in the phylogenetic tree, there is a degree of convergent evolution in the respective taxa.

## Conclusions

In the reef-building polychaete *Sabellaria alveolata,* the entire body is covered by a thin and delicate cuticle characterized by the absence of layers of parallel collagen fibers. Most likely this absence is responsible for the lack of differentiation between the various body regions including the branchiae. All appendages, including the branchiae, bear receptor cells and, as such, are sensory. The opercular papillae with their tufts of receptor cells and lack of motile cilia resemble parapodial cirri of other polychaetes, and their main function may be sensory. In contrast, the tentacular filaments have a triple function; sensing and collecting and transporting particles. A similarity to branchiae can be excluded since blood vessels are absent. Besides their distinctive ciliation pattern, the most conspicuous morphological feature of these filaments is their cell-free endoskeletal cartilaginous structure enclosed by ECM. The palps represent typical grooved palps with two coelomic cavities and a single blind-ending blood vessel [4, 64, 65]. They are adapted for collecting and transporting particles as well as for sensing. The palps are structurally similar to those of other sabellariids studied [15, 16]. A revised classification of polychaete branchiae is suggested; the branchiae of *S. alveolata* belong to the most common type comprising musculature, coelom, and blood vessels. So far this type only occurs in a subclade of Sedentaria. These branchiae are highly vascularized and equipped with numerous blood spaces extending deep into the basal region of the epidermal cells resulting in short diffusion distances between blood and environment of 150–400 nm.

## Methods

### Material

Adult specimens of *Sabellaria alveolata* (Linnaeus, 1767) were collected at Saint-Efflam (Brittany, France) in summer 2017 (*n* > 30). Additional specimens were collected from reefs at Mont St. Michel Bay (Normandy, France; collected and fixed by Dr. Larisse Faroni-Perez; n ≈ 20). Very small parts of the reefs were dislodged and reared in the laboratory under running seawater until further processing. Animals were removed from their tubes by carefully crashing the walls. Only intact individuals were used for fixation, which occurred at the Marine Biological Station of Roscoff (Brittany, France).

### Electron microscopy, semithin sections, and histology

The smallest adult individuals were chosen for fixation (*n* = 20). Specimens from St. Efflam were relaxed in 8% magnesium chloride (MgCl_2_ x 10H_2_O; 15 min) isotonic with seawater, and fixed immediately in a phosphate-buffered (0.075 M) solution of picric acid, paraformaldehyde, and glutaraldehyde adjusted to the appropriate osmolality with sucrose (SPAFG, [93]) for 2 h at 4 °C. Specimens from Mont St. Michel were fixed in 2.5% glutaraldehyde in 0.05 M phosphate buffer with 0.3 M NaCl. After five rinses in the appropriate buffer for 10 min each, specimens were stored in the same buffer containing 0.05% NaN_3_ at 4 °C. After a few days, they were further processed in the lab of the Zoology department, Osnabrueck. Specimens were then post-fixed in 1% OsO_4_ (phosphate-buffered) for 1 h at 4 °C. After being washed for 5 min in either 0,075 M buffer adjusted with sucrose or 0.05 M buffer adjusted with NaCl, samples were dehydrated using an Ethanol series (30% for 5 min at 4 °C, 50% for 5 min at 4 °C, 70% for 10 min 4 °C, 80% for 10 min at 4 °C, 95% for 10 min at 4 °C, 95% for 10 min at RT, and finally, 2 × 100% for 10 min at RT).

About 10 specimens were chosen for scanning electron microscopy (SEM). They were then critical-point dried using CO_2_(l) (CPD 030, Bal-Tec, Präffikon, Switzerland), mounted with adhesive tabs on aluminum stubs, coated with platinum/iridium (sputter coater K575, Emitech, Montigny de Bretonneux, France) and examined with a Zeiss Auriga (Oberkochen, Germany) scanning electron microscope.

For transmission electron microscopy (TEM) and semithin sectioning histology, the smallest specimens available were chosen. They were dissected into smaller parts. These were transferred into a solution of ethanol and the intermedium propylene oxide (100% EtOH: propylene oxide 1:1, 2 × 30 min) followed by pure propylene oxide (4 × 15 min). This solution was replaced by mixtures of the intermedium and the embedding medium starting with propylene oxide: Araldite/Epon (PolyBed 812) 3:1 for 6 h, followed by 2:1 (12 h) and finally 1:1 (12 h). The intermedium was then allowed to evaporate overnight. Before final embedding, specimens were transferred into drops of fresh Araldite/Epon for 5 min at 60 °C. After two repeats, specimens were placed in embedding molds. Polymerization was conducted at 60 °C for 72 h. Eight specimens were either cut in series of semithin (1 μm) or ultrathin sections through the respective appendages (70 nm) using diamond knives (Diatome, Biel, Switzerland) and UC6 or UC7 Leica ultra-microtomes (Wetzlar, Germany). One specimen of these was cut in a series of semithin sections with subsets of about 20 ultrathin sections taken every 10 μm.

The series of ultrathin sections were placed on single-slot grids coated with pioloform support films, then contrasted at 20 °C with 2% uranyl acetate (30 min) and 0.5% lead citrate (20 min) in a nanofilm surface analysis ultrastainer (Göttingen, Germany). Finally, the sections were examined with Zeiss EM 902A and Zeiss Libra 120 transmission electron microscopes (Oberkochen, Germany). Images were recorded using CCD cameras (Image SP®, 4 k, Mohrenweis, Germany).

A series of semithin sections was obtained by covering the trimmed block with glue (Pattex®, Henkel, Germany, diluted 3:1 with xylene [94];). Sections were collected on glass slides, stained with toluidine blue (0.5% toluidine blue in a 1% aqueous solution of borax for 15–30s at 60 °C), rinsed with H_2_O, fixed with 5% ammonium molybdate tetrahydrate ((NH_4_)_6_Mo_7_O_24_ × 4 H_2_O) and mounted with Entellan mounting medium. Alternatively, sections were stained in a 0.1% solution of toluidine blue for approximately 25 min; further processing was as described above. Pictures were taken with a DMLS light microscope, Leica, Wetzlar, Germany, equipped with a Progress Gryphax® CCD camera (Jenoptik, Jena, Germany) and Gryphax software.

### Immunohistochemistry

For confocal laser scanning microscopy (cLSM), about 5 small animals were fixed in 4% paraformaldehyde in phosphate-buffered saline (PPS: 140 mM NaCl, 6.5 mM KCl, 2.5 mM Na_2_HPO_4_, 1.5 mM KH_2_PO_4_, 12% sucrose, pH 7.4) at 4 °C and 2.5 h. After fixation, specimens were rinsed in PBS and stored in the same buffer containing a few crystals of NaN_3_ to avoid growth of bacteria and fungi. Either whole mounts or dissected specimens were used. Prior to immunolabelling, specimens were incubated with PBT (9 ml PBS + 1 ml 1% Triton X-100) containing 6% BSA (bovine serum albumin) for 1 h and finally incubated with the primary antibody for 2–4d at 4 °C. Primary antibody was mouse anti-acetylated α-tubulin (monoclonal, clone 6–11-B-1; Sigma-Aldrich, Heidelberg, Germany, dilution 1:1000 in PBT). Following several washes (3x in PBT, 20 min each), the secondary antibody was applied for 2–3d at 4 °C (goat anti-mouse, Cy2 conjugated, Dianova, Hamburg, Germany, dilution 1:200). After being rinsed three times for 10 min in PBS, specimens were mounted in Fluoromount (Southern Biotech, Birmingham, USA). Observations were made with a Zeiss Pascal 5 confocal laser scanning microscope (Zeiss, Jena, Germany). Z-stacks are displayed as maximum projections if not stated otherwise.

### Analysis and 3D reconstruction

All images were further processed using either Photoshop® or Affinity Photo® to adjust brightness, contrast, and size, and Illustrator® or Affinity Designer® for assembling and labeling the plates. 3-D reconstructions were made from a series of semithin sections comprising approximately 400 sections. Micrographs were taken in intervals of 5 μm and blood vessels, coelomic cavities, other tissues were segmented with ImageJ/FIJI [95, 96] using the TrakEM2 Plugin [97]. After alignment of the sections, the 3-D model was calculated with the 3DViewer Plugin [98]. Measurements have been taken on micrographs from randomly selected sections with at least 10 replicates on different sections if not stated otherwise.

## Supplementary Information

**Additional file 1: Figure S1**. Schematic 3D Model of an exemplified mid-body branchia in *Sabellaria alveolata*. Generated from a series of approximately 400 semithin sections (1 μm each) utilizing every 5th section for reconstruction. Only branchial coelom shown. Color code: green: epidermis, red: blood vessels, blue: coelom. **A, C, E:** view from exterior, **B, D, F:** view from inner side. **A, B:** Reconstruction showing epidermis, coelom and blood vessels. **C, D**: Coelom and blood vessels, epidermis omitted. **E, F:** Blood vessels only, epidermis and coelom omitted. Same scale in A-F.

**Additional file 2: Figure S2**. Slightly oblique longitudinal section through basal region of branchia in *Sabellaria alveolata* to show overall organization of branchiae on a single TEM section with comparatively high resolution. Note the high amount of blood spaces in the basal part and their continuous decrease in the upper part of the branchia. Due to oblique direction of the section the coelom appears larger than it is on cross sections (compare Fig. 8D). Coelomic lining represented by myoepithelial cells. Landscape view: Branchia oriented with dorsal to the right and median up. Panorama taken out of 34 single images made at 3000x magnification and stitched with Affinity Photo®. Background modified to ensure a more homogenous appearance. Image size at 354pixel/inch: 100 by 40 cm.

**Additional file 3 Video clip of 3D reconstruction**. The video shows a rotation of the branchia reconstructed in Fig. S1 with the same color code and subsequently omitting epidermis and coelom. Color code: green: Epidermis, red: blood vessels, blue: coelom.

## Data Availability

The material of *Sabellaria alveolata* (embedded blocks, semi-thin sections, and ultrathin sections) are stored at the Department of Zoology and Developmental Biology at the University of Osnabrueck. All images taken are stored in the database Omero hosted at the University of Osnabrueck. These images are available from the corresponding author on reasonable request.

## Abbreviations

3D: Three dimensional
AC: Accessory centriole
AFS: Abfrontal side
BB: Basal body
BC: Blood cell (hemocyte)
BP: Basal plate
BR: Branchia
BRCOE: Branchial coelom
BRN: Branchial nerve
BS: Blood space
BV: Blood vessel
CB: Ciliary band
CC: Ciliated cell
CCB: Curved ciliary band
CG: Group of long cilia
CL: Cilium
cLSM: Confocal laser scanning microscopy
COE: Coelom
CT: Connective tissue
CU: Cuticle
DBV: Dorsal blood vessel
DLM: Dorsal longitudinal musculature
DN: Dorsal nerve
ecm: ECM
ECM: Extracellular matrix
FS: Frontal side
G: Gut
GC: Glandular cell
HB: Heart body
HC: Hyaline cartilage structure
IBRV: Inner branchial vessel
IF: Intermediate filaments
LM: Longitudinal muscle
LY: Lysosome
MB: Multivesicular body
MF: Myofilaments
MI: Mitochondrion
MO: Mouth
MU: Musculature
MV: Microvillus
N: Nerve
NC: Nucleus
NEU: Neuropodium
NOT: Notopodium
OBRV: Outer branchial vessel
OP: Opercular papilla
PA: Opercular palea
PP: Palp
PT: Peritoneum
RC: Receptor cell
SC: Sensory cilium
SCB: Straight ciliary band
SEM: Scanning electron microscopy
SJ: Septate junction
SN: Segmental nerve
SUC: Supportive cell
TEM: Transmission electron microscopy
TF: Tentacular filament
V: Vesicle
vlm: Ventral longitudinal muscle
vnc: Ventral nerve cord
ZA: Zonula adherens
