# Supplementary figures and images for: Ultrastructure and functional morphology of the appendages in the reef-building sedentary polychaete Sabellaria alveolata (Annelida, Sedentaria, Sabellida)
Source: BMC Zool. 2021 Mar 9;6:5. doi: 10.1186/s40850-021-00068-8 (PMC10127387; doi:10.1186/s40850-021-00068-8)

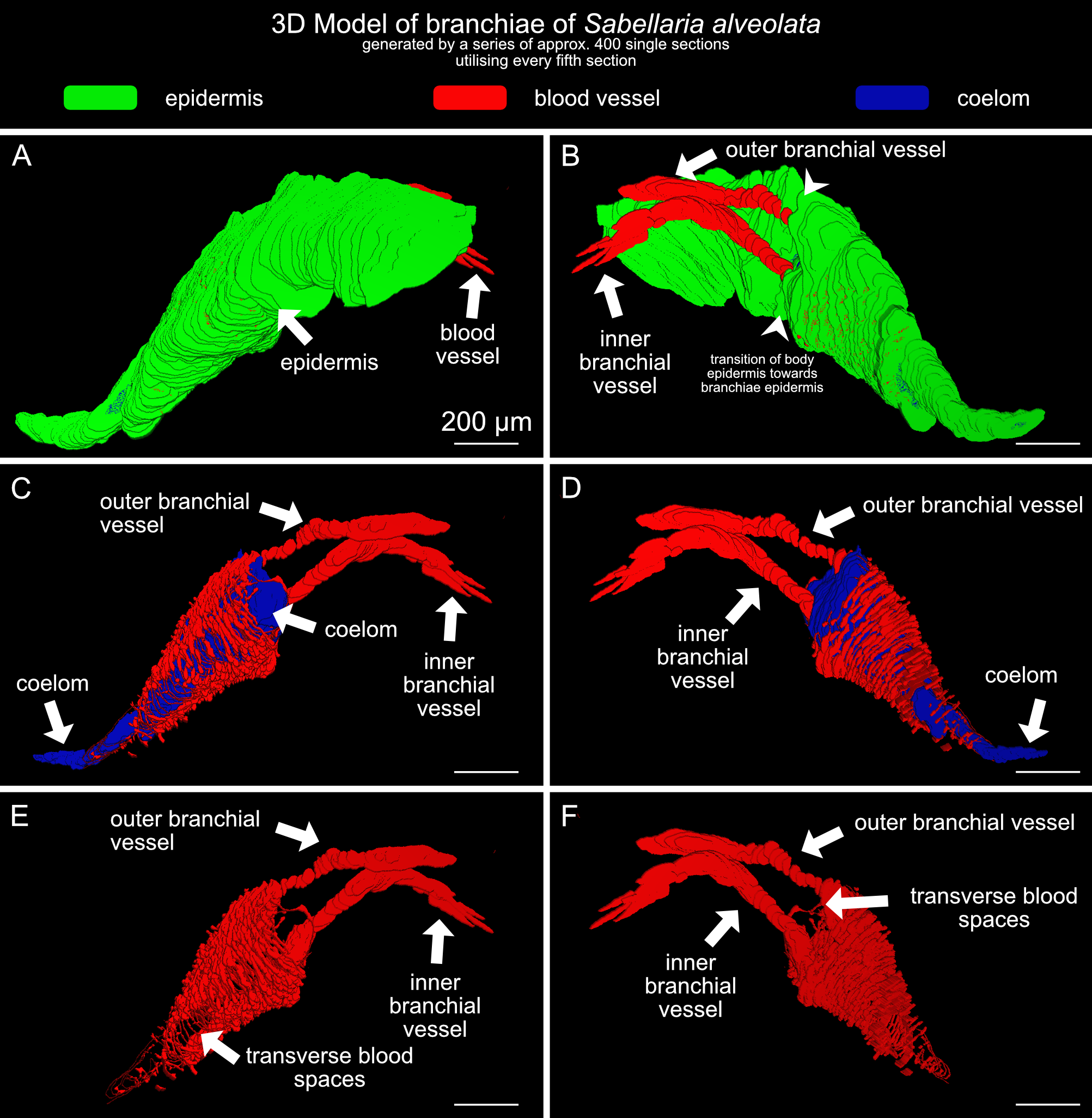

Supplement: Supplementary file 1 — Additional file 1: Figure S1. Schematic 3D Model of an exemplified mid-body branchia in Sabellaria alveolata. Generated from a series of approximately 400 semithin sections (1 μm each) utilizing every 5th section for reconstruction. Only branchial coelom shown. Color code: green: epidermis, red: blood vessels, blue: coelom. A, C, E: view from exterior, B, D, F: view from inner side. A, B: Reconstruction showing epidermis, coelom and blood vessels. C, D: Coelom and blood vessels, epidermis omitted. E, F: Blood vessels only, epidermis and coelom omitted. Same scale in A-F. [file 40850_2021_68_MOESM1_ESM.tiff]
